# UniMap: Type‐Level Integration Enhances Biological Preservation and Interpretability in Single‐Cell Annotation

**DOI:** 10.1002/advs.202410790

**Published:** 2025-02-27

**Authors:** Haitao Hu, Yue Guo, Fujing Ge, Hao Yin, Hao Zhang, Zhesheng Zhou, Fangjie Yan, Qing Ye, Jialu Wu, Ji Cao, Chang‐Yu Hsieh, Bo Yang

**Affiliations:** ^1^ Institute of Pharmacology and Toxicology Zhejiang Province Key Laboratory of Anti‐Cancer Drug Research College of Pharmaceutical Sciences Zhejiang University Hangzhou 310058 China; ^2^ Polytechnic Institute of Zhejiang University Zhejiang University Hangzhou 310015 China; ^3^ College of Pharmaceutical Sciences Zhejiang University Hangzhou Zhejiang 310058 P. R. China; ^4^ The Innovation Institute for Artificial Intelligence in Medicine Zhejiang University Hangzhou 310018 China; ^5^ Engineering Research Center of Innovative Anticancer Drugs Ministry of Education Hangzhou 310000 China; ^6^ Center for Medical Research and Innovation in Digestive System Tumors Ministry of Education Hangzhou 310020 China; ^7^ School of Medicine Hangzhou City University Hangzhou 310015 China

**Keywords:** batch effects, biological conservation, multi‐selective adversarial networks, single‐cell annotation, type‐level integration

## Abstract

Integrating single‐cell datasets from multiple studies provides a cost‐effective way to build comprehensive cell atlases, granting deeper insights into cellular characteristics across diverse biological systems. However, current data integration methods struggle with interference in partially overlapping datasets and varying annotation granularities. Here, a multiselective adversarial network is introduced for the first time and present UniMap, which functions as a “discerner” to identify and exclude interfering cells from various data sources during dataset integration. Compared to other state‐of‐the‐art methods, UniMap emphasizes type‐level integration and proves to be the best model for preserving biological variability, achieving noticeably higher accuracy in single‐cell automated annotation under various circumstances. Additionally, it enhances interpretability by revealing shared and domain‐specific cell types and providing prediction confidence. The efficacy of UniMap is demonstrated in terms of identifying new cell types, creating high‐resolution cell atlases, annotating cells along developmental trajectories, and performing cross‐species analysis, underscoring its potential as a robust tool for single‐cell research.

## Graphical abstract

UniMap introduces a novel multiselective adversarial network that functions as a discerner in single‐cell dataset integration. It enables type‐level integration, ensuring accurate cell type annotation across diverse biological contexts while preserving shared and domain‐specific features. UniMap outperforms by maintaining biological variability and enhancing interpretability, making it a powerful tool for single‐cell research.

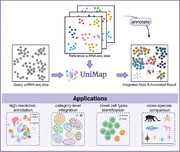

## 1 Introduction

Single‐cell sequencing technologies, with their unprecedented resolutions, have fundamentally revolutionized our understanding of basic biological processes and various disease states, such as cell differentiation,^[^ ^1^, ^2^ ^]^ cell communication,^[^ ^3^ ^]^ and the tumor microenvironments.^[^ ^4^ ^]^ Recently, several cutting‐edge large‐scale studies have aimed to create comprehensive reference maps of all human cells, other species, or specific disease contexts.^[^ ^5^, ^6^, ^7^ ^]^ However, due to the high costs associated with such studies, a more economical approach involves integrating existing datasets to create more comprehensive atlases for specific, less‐studied biological context.

The major issue is how to effectively align Single cell RNA sequencing (scRNA‐seq) data from different domains, given the substantial batch effects across datasets acquired from diverse studies, sequencing methods, and sample origins.^[^ ^8^ ^]^ Current data integration methods aim to align various datasets within a low‐dimensional space, while enabling automated cell annotation for unannotated datasets through label transfer in the shared feature space, thereby advancing research in the field. For example, classic machine learning integration methods, such as Harmony,^[^ ^9^ ^]^ Seurat v5,^[^ ^10^ ^]^ fastMNN,^[^ ^11^ ^]^ Scanorama,^[^ ^12^ ^]^ and BBKNN,^[^ ^13^ ^]^ use strategies like soft clustering or mutual nearest neighbor techniques to integrate similar cells across different datasets. Subsequently, deep learning models based on auto‐encoders or variational auto‐encoders, including scVI,^[^ ^14^ ^]^ scANVI,^[^ ^15^ ^]^ trVAE,^[^ ^16^ ^]^ Scalex,^[^ ^17^ ^]^ and scPoli,^[^ ^18^ ^]^ have demonstrated strong capabilities in reducing batch effects in low‐dimensional latent spaces. Additionally, TOSICA^[^ ^19^ ^]^ employs a transformer structure with pathway‐based attention scoring, providing biologically interpretable insights. Furthermore, models employing graph neural networks (GNN) and generative adversarial networks (GAN), such as scGraph,^[^ ^20^ ^]^ scGen,^[^ ^21^ ^]^ Dmatch,^[^ ^22^ ^]^ exhibits advantages in generation or interpretability.

However, traditional models may struggle with the “data drift problem”^[^ ^23^ ^]^ when applied in real‐world scenarios as the scale and complexity of single‐cell studies increase. In practice, cell types in different datasets often partially overlap, meaning both query and reference datasets contain unique cell types (interfering cells and cell types) not present in each other. Most current prominent methods typically assume a fully shared label space across datasets, without considering domain‐specific cell types. Consequently, these models often suffer from overcorrection and reduced predictive accuracy when faced with partially overlapping datasets, especially when there are only few shared cell types between the reference atlas and the query dataset.

Currently, two of the latest annotation tools, ssSTACAS^[^ ^24^ ^]^ and Portal^[^ ^25^ ^]^ sought to address the challenge of partially overlapping datasets with the aim of preserving biological variance as much as possible while eliminating batch effects to improve annotation performance. ssSTACAS utilizes an anchor‐based machine learning algorithm that removes certain anchor relationships by verifying if the closest cells from different datasets in the Principal component analysis (PCA) space have matching labels before constructing the decision tree. While this strategy effectively mitigates some impact from interfering cells, it may inadvertently exclude similar cells with different subtypes when applied to large‐scale atlases comprising numerous cell types. Moreover, Portal uses a domain translation network to create dataset‐specific feature spaces and employs discriminators with a score threshold to identify domain‐specific and shared cell types. However, due to the lack of label information, Portal performs poorly in capturing subtle cell subtype differences, which limits its application in precise annotation using high‐resolution atlases.

To address the aforementioned issues, we develop **UniMap**, an end‐to‐end supervised framework designed for atlas‐level data integration and high‐resolution cell annotation. UniMap utilizes a multiselective adversarial network^[^ ^26^, ^27^ ^]^ with type‐specific discriminators to achieve type‐level integration across domains, rather than global integration. Additionally, we introduce a dynamically learned weight $w_{r}^{\mathrm{ct}}$ to explicitly identify shared and unique cell types within partially overlapping datasets. These strategies enable UniMap to selectively integrate shared cell types while preserving domain‐specific biological information and avoiding overcorrection. Furthermore, under a supervised setting, UniMap retains subtle cell subtype information from reference atlases, making it suitable for creating high‐quality atlases and performing high‐resolution cell annotation.

We demonstrate that UniMap is the best model for preserving biological variability in data integration and cell annotation tasks through comparisons with other state‐of‐the‐art (SOTA) methods. In the context of partially overlapping datasets, UniMap achieves the most balanced performance in terms of biological conservation and batch correction. It selectively integrates shared cell types while preserving the intrinsic features of interfering cells, thereby avoiding overcorrection. Furthermore, UniMap better preserves subtle differences between cell subtypes, allowing for high‐resolution predictions in coarse‐grained query datasets. In the case of creating PBMC atlases for severe myasthenia gravis (MG) patients, UniMap proves to be a powerful tool for integrating imperfect atlases, creating complete, high‐resolution maps by finely merging both shared and unique cell types from reference datasets. Crucially, the weight knowledge learned by UniMap facilitates multiscale interpretability analyses at both the cell and type levels and enhances the reliability of downstream analyses by filtering out low‐quality predictions. These characteristics support its applicability to more complex scenarios, such as cross‐species comparison, improving our understanding of conservation and differences between species at single‐cell resolution.

## 2 Results

### 2.1 Method Overview

**Figure 1:**
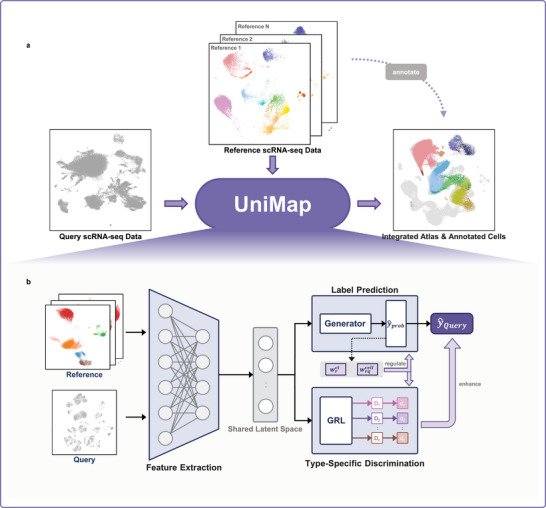
Overview of UniMap. a) UniMap utilizes annotated scRNA‐seq datasets to integrate and annotate unlabeled query data by eliminating batch effects and preserving biological information. b) Three modules of UniMap. The feature extraction module maps the gene expression information of reference and query data into a shared latent space while preserving the biological signals. The label prediction module trains a classifier using the labels of the reference data and calculates a cell type weight ($w_{r}^{\mathrm{ct}})$ and a cell weight ($w_{q}^{\mathrm{cell}}$) through SoftMax output. The type‐specific discrimination module includes a gradient reversal layer (GRL) and *d* identical discriminators (where *d* is the number of cell types). Each discriminator is used to identify the source of cells within a specific cell type, utilizing $w_{r}^{\mathrm{ct}}$ and $w_{q}^{\mathrm{cell}}$ to facilitate selective data mixing and reduce interference.

UniMap is a multifunctional tool that integrates expert‐curated reference maps to create a comprehensive cell atlas while simultaneously providing automatic annotation for unlabeled single‐cell datasets (**Figure** **1**a). By using a supervised learning approach, UniMap learns to project expression matrices derived from various datasets to a shared cell representation space and uses these features for label prediction.

UniMap consists of three components: a feature extraction module, a label prediction module, and a type‐specific discrimination module (Figure 1b). The initial step, feature extraction, involves transforming gene expression matrices and batch information into low‐dimensional representations, where shared cell types across different domains are integrated. These representations are then fed into the label prediction module. Notably, during parameter updates, this module generates two essential weights: a cell type weight ($w_{r}^{\mathrm{ct}}\in {\left[0,1\right]}^{d}$, where ***d*** is the number of cell types contained in the reference data) and a cell weight ($w_{\mathrm{rq}}^{\mathrm{cell}}\in {\left[0,1\right]}^{m+n}$, where ***m*** and ***n*** are the numbers of cells in the reference and query data, respectively). Specifically, $w_{r}^{\mathrm{ct}}$ indicates the importance of different cell types in the reference data, with low values indicating potentiallyinterfering cell types. Moreover, $w_{\mathrm{rq}}^{\mathrm{cell}}$ reflects the importance of each cell from both the reference and query sources, with low values indicating low‐contribution cells in the integration process.

The type‐specific discrimination module incorporates a gradient reversal layer (GRL)^[^ ^28^ ^]^ and ***d*** identical discriminators, which are functionally coupled with $w_{r}^{\mathrm{ct}}$ and $w_{\mathrm{rq}}^{\mathrm{cell}}$. The GRL inverts the signs of gradients during backpropagation, allowing the discriminators to progressively improve their ability to differentiate between the reference and query data sources during training. This mechanism operates like a “generative adversarial network”,^[^ ^29^ ^]^ ultimately integrating data from various sources into a shared low‐dimensional space. Each discriminator ***d***_***j***_ aims to ascertain whether a cell categorized as ***j*** or predicted as ***j*** originates from the reference or query source. When calculating the loss function, ***d***_***j***_ considers its corresponding $w_{r}^{\mathrm{ct}}\left[j\right]$. This strategy ensures that UniMap excludes discriminators that identify interfering cell types, thereby minimizing the impact of interfering cells and facilitating integration in partially overlapping datasets. Further details are provided in the Experimental Section.

### 2.2 UniMap Outperforms the SOTA Methods in Terms of Integration and Annotation

We assessed the integration and annotation performance of UniMap by comparing it with other SOTA approaches obtained from recent benchmark studies.^[^ ^30^, ^31^ ^]^ Our comparison approaches spanned deep learning models such as CODE‐MMD,^[^ ^32^ ^]^ CODE‐ADV,^[^ ^32^ ^]^ Scalex,^[^ ^17^ ^]^ scPoli,^[^ ^18^ ^]^ and Portal^[^ ^25^ ^]^ along with other machine learning methods such as Harmony,^[^ ^9^ ^]^ Seurat v5,^[^ ^10^ ^]^ BBKNN,^[^ ^13^ ^]^ and Ingest.^[^ ^33^ ^]^ Additionally, we conducted comparisons with scGPT,^[^ ^34^ ^]^ a prominent single‐cell large language model (LLM) in fine‐tuning setting.

Our evaluation focused on quantitatively assessing methods in batch correction and biological conservation aspects. We utilized several metrics to evaluate batch correction efficacy, including graph connectivity (GC), the graph integration local inverse Simpson's index (Graph iLISI), the k‐nearest neighbor batch effect test (kBET), and the modified average silhouette width of the batch (Batch ASW). To assess the methods' ability to conserve biological variations, we calculated seven additional metrics: the adjusted Rand index (ARI), the graph cell type‐local inverse Simpson's index (Graph cLISI) score, the average silhouette width (ASW), the isolated label score‐ASW (Label ASW), the isolated label F1, the normalized mutual information (NMI), the overcorrection score and the annotation accuracy. We then computed the average rankings for each evaluation metric to represent the overall performance of the models. On the other hand, we also evaluated the model's running time and peak memory usage, which demonstrated that UniMap maintains reasonable computational overhead (Figure S1, Supporting Information).

We conducted our evaluation experiment on a labeled dataset consisting of peripheral blood mononuclear cells (PBMCs) acquired from patients with common variable immunodeficiency (CVID).^[^ ^35^ ^]^ The dataset comprises 35699 cells distributed across 9 cell types that were derived from samples obtained from 6 donors. Three batches of data (1 normal lung tissue sample and 2 emphysema lung tissue samples) were chosen as labeled reference data to annotate other three batches of query data (2 normal lung tissue samples and 1 emphysema lung tissue sample). Furthermore, to evaluate the various models on partially overlapping datasets, we excluded plasma cells from the reference data and dendritic cells from the query data, designating them as interfering cell types.

**Figure 2:**
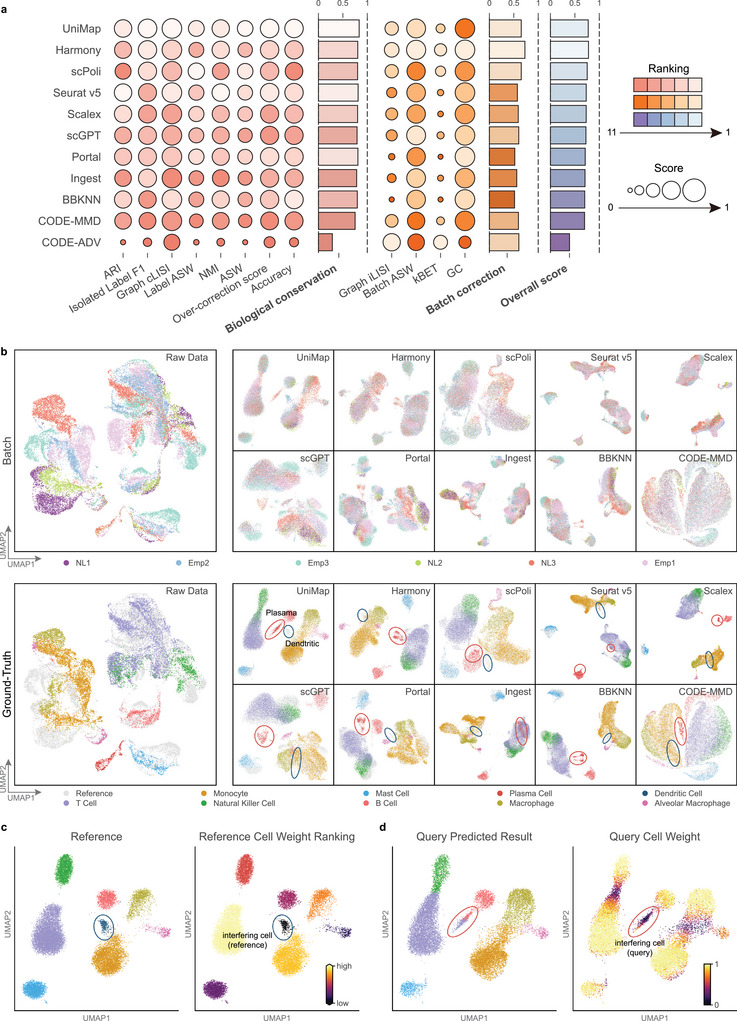
Benchmark study in partially overlapping datasets. a) Overview of all benchmark models (UniMap, Harmony, scPoli, Seurat v5, Scalex, scGPT, Portal, Ingest, BBKNN, and CODE‐MMD) by overall score (purple) based on the PBMC CVID dataset. Metrics are divided into biological conservation (red) and batch correction (orange). Overall scores are computed using the average of all individual metrics. All scores are normalized to a range of 0 to 1, with the higher values indicating better performance. b) UMAP plots showing the raw data and the integration results of benchmark models, colored by batches (upper) and ground‐truth labels (lower). Interfering cells in the reference and query are highlighted with blue circles and red circles, respectively. c) UMAP plots of the integrated reference data by UniMap, colored by ground‐truth labels and reference cell weight rankings. d) UMAP plots of the integrated query data by UniMap, colored by predicted labels and query cell weights.

On the basis of the benchmarking results, we found that UniMap demonstrated most balanced performance, ranking 1st in biological conservation and 3rd in batch correction (**Figure** **2**a). Other models either underperformed in biological conservation, such as Harmony and scPoli, or were less effective in batch integration, like Portal and Seurat v5. Unlike traditional methods, UniMap achieves type‐level integration by the weighting mechanism and multi‐selective discriminators. This allows it to selectively integrate shared cell types across different domains while discerning interfering cell types. As shown in Figure 2c,d, UniMap accurately assigned lower weights to interfering cell types (dendritic cells in the reference datasets and plasma cells in the query) and higher weights to shared cell types.

Furthermore, UniMap effectively preserved the biological information of interfering cells during batch correction, as both types of interfering cells were identified as separate clusters without mixing with other cell types (Figure 2b). In contrast, models like Seurat v5, scPoli, and Ingest struggled to distinguish query interfering cells (plasma cells), often misclassifying them as B cells or T cells. Additionally, models such as Scalex, BBKNN, Portal, and scGPT mixed interfering cells (dendritic cells) with others, thus reducing the quality of the reference atlas (Figure S2, Supporting Information). We quantified this aspect using the over‐correction score^[^ ^17^ ^]^ proposed by Xiong et al., which measures the percentage of cells with inconsistent cell types within each cell's neighborhood. Our results indicated that UniMap performed the best in avoiding overcorrection when handling partially overlapping datasets (Figure 2a). Altogether, UniMap excelled at integrating partially overlapping datasets, achieving accurate predictions while preserving biological differences, making it a robust tool for dataset integration and cell annotation.

### 2.3 UniMap Delineates Reference‐Guided Bone Marrow Trajectories

**Figure 3:**
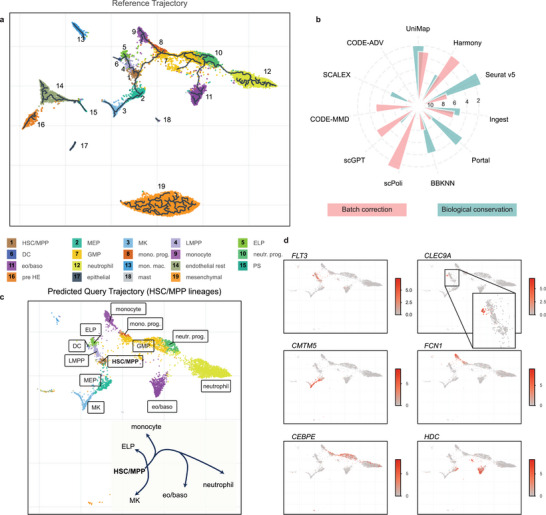
Localization of query cells along a trajectory of BM reference. a) UMAP plots showing the cell population with ground‐truth labels. Black lines indicate predicted developmental trajectories originating from HSC/MPPs, as inferred by Monocle 3 pseudo time analysis. b) Overview of all benchmark models by biological conservation rank (pink) and batch correction rank (green) based on BM dataset, where sector size corresponds to method ranking. c) The inferred query cells preserve branching within the HSC/MPPs lineages, placing terminally differentiated states on the ends. d) Expression of lineage marker genes (*FLT3* for *ELP*, *CMTM5* for MK, *CEBPE* for neutrophil, *CLEC9A* for DC, *FCN1* for mature monocytes, and *HDC* for eo/baso). Cells colored by log‐normalized expression of gene.

To further evaluate UniMap's ability to capture intrinsic biological divergence between datasets, we tested its capability to preserve the gradual changes in cell states, particularly in the context of cellular differentiation and cell cycle dynamics. We used a scRNA‐seq dataset derived from Bone Marrow (BM) organoids, which includes 31040 cells collected across five batches and representing 19 author‐defined cell types.^[^ ^36^ ^]^ Three batches were designated as reference data, and Monocle3^[^ ^37^ ^]^ trajectory analysis was employed to elucidate the developmental relationships between progenitor and differentiated cell populations. Notably, our analysis revealed that the hematopoietic stem cell and multipotent progenitor (HSC/MPP) compartment gave rise to five distinct developmental trajectories, corresponding to megakaryocytic (MK), early lymphoid progenitor (ELP), monocyte, eosinophil/basophil (eo/baso), and neutrophil lineages. This reference atlas contains two forms of annotation for downstream query inference: discrete cell types and positions along differentiation gradients (**Figure** **3**a).

Subsequently, we mapped the remaining 2 batches onto the reference atlas (Figure 3c). The quantitative assessment of mapping performance demonstrated that UniMap ranked 1st and 3rd in terms of biological conservation and batch correction, respectively (Figure 3b). UniMap maintained its superior performance while achieving an optimal balance between preserving biological signals and mitigating technical variations. Moreover, transitions from HSC/MPPs to differentiated types were characterized by gradual changes in canonical marker genes: *FLT3* for ELP, *CMTM5* for MK, CEBPE for neutrophil, CLEC9A for DC, FCN1 for mature monocytes, and HDC for eo/baso (Figure 3d).

### 2.4 UniMap Supports Higher‐Resolution Cell Annotation via High‐Quality Annotated Atlases

Another challenge encountered when integrating single‐cell data is the inconsistency exhibited by the cell label resolutions across different studies. High‐resolution annotations are essential for revealing biological mechanisms in specific contexts, such as for constructing tissue‐specific or disease‐specific single‐cell atlases, which require detailed categorizations of cell subtypes, potentially involving millions of cells.^[^ ^38^, ^39^ ^]^ However, many studies provide only low‐resolution cell labels. A practical solution is to use existing high‐quality atlases for precise automated cell annotation of queried datasets. Nonetheless, this approach poses a significant challenge to the model's ability to preserve biological information.

**Figure 4:**
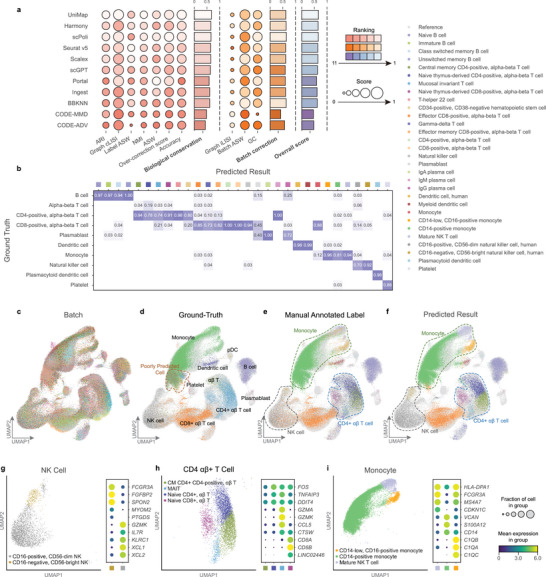
High‐resolution cell annotation with UniMap. a) Overview of all benchmark models by overall score based on PBMC MG datasets. Metrics are divided into biological conservation (red) and batch correction (orange). Overall scores are computed using the average of all individual metrics. All scores are normalized to a range of 0 to 1, with the higher values indicating better performance. Isolated Label F1 and kBET metrics were not calculated in this case due to computational overhead constraints. b) Confusion matrix comparing predicted labels by UniMap and ground‐truth labels from the original PBMC COVID‐19 dataset publication. The color intensity indicates the proportion of cells in confusion matrix *C_ij_* known to be cell type *i* and predicted to be cell type *j*. c–f) UMAP plots of the integration results by UniMap, colored by batches, ground‐truth labels (10 cell types), manual annotated labels (18 cell types), and predicted labels (30 cell types). g–i) Left: UMAP plots of the integration results by UniMap for cell populations with ground‐truth labels of NK cells, CD+ αβ T cells, and monocytes. Right: Dot plots showing expression of differentially expressed genes among the predicted subtypes of three cell populations, with the color and size representing normalized gene expression and the percentage of cells expressing a given gene, respectively.

To evaluate UniMap's ability for high‐resolution annotation, we used a PBMC COVID‐19 dataset^[^ ^40^ ^]^ with 201880 cells acquired from 40 individuals as a high‐resolution reference atlas to annotate another PBMC COVID‐19 dataset,^[^ ^41^ ^]^ which included samples derived from 15 individuals and was labeled with low‐resolution cell annotations. The results showed that UniMap successfully integrated data from different batches and precisely subdivided the query cells into 30 subtypes, whereas they were originally annotated with only 10 cell types (**Figure** **4**a–f). This improvement is attributed the assignment of a specific discriminator for each distinct cell type in the reference, enabling the model to learn subtle differences between subtypes more effectively. In contrast, most other models failed to retain such fine biological distinctions, leading to reduced quality of reference atlas and incorrect annotations, as evidenced by UMAP plots and annotation results (Figures S3 and S5, Supporting Information).

To facilitate a rigorous comparison of predictive outcomes across models, we manually annotated the query data following the high‐resolution annotation protocols established in the reference dataset, with the cell subtype annotations supported by their characteristic marker genes (Table S1, Supporting Information). Based on these new fine‐grained annotations, we quantitatively assessed the integration and higher‐resolution annotation results of UniMap and other models. The results demonstrate that UniMap ranked 1st in both biological conservation and annotation accuracy, highlighting its potential in leveraging high‐quality single‐cell atlases for future applications (Figure S5, Supporting Information).

Moreover, we developed and implemented the adjusted Shannon diversity index as a novel quantitative metric to evaluate model performance in high‐resolution annotation tasks. This metric assesses the proportion of different low‐resolution ground‐truth labels associated with each predicted cell subtype. A higher value indicates that the ground‐truth labels for each predicted cell subtype are more uniform, signifying more reliable predictions. For example, an ideal prediction entails that a cell population identified as a specific subtype (e.g., CD14‐positive or CD16‐positive monocyte cells) matches the ground‐truth labels (e.g., monocytes) without any admixture from other cell types (e.g., B cells or T cells). In this regard, UniMap ranked 1st, demonstrating its strong capability in capturing subtle biological information (Figure S6, Supporting Information).

Furthermore, we examined the gene markers in various cell subclusters to validate the UniMap predictions. The NK cell population, was split into two major subtypes: CD16‐positive CD56‐dim NK cells expressing *GZMK*, *IL7R*, *KLRC1*, *XCL1*, and *XCL2* and CD16‐negative CD56‐bright NK cells with increased expressions of *FCGR3A*, *FGFBP2*, and *SPON2* (Figure 4g). These genes aligned with the findings reported by Adeline Crinier^[^ ^42^ ^]^ and colleagues. The CD4‐positive αβ T cell population was categorized into four main types: mucosal‐associated invariant T cells (MAIT) expressing the cytotoxic effector granules *GZMA* and *GZMK*, which are typical of MAIT cells;^[^ ^42^, ^43^ ^]^ naive thymus‐derived CD8‐positive αβ T cells with highly expressed marker genes such as *CD8A* and *CD8B*; and central memory CD4‐positive αβ T cells and naive thymus‐derived CD4‐positive αβ T cells, which displayed similar gene expression profiles, with slight differences in genes such as *FOS*, *TNFAIP3*, and *DDIT4* (Figure 4h). The monocyte population was primarily predicted to include CD14‐low CD16‐positive monocytes, CD14‐positive monocytes, and mature NK T cells. Among these, CD14‐low CD16‐positive monocytes were characterized mainly by the expression of three complementary component 1q (C1Q) genes (*C1QA*, *C1QB*, and *C1QC)*, which play important roles in immune recognition in both adaptive and innate immunity;^[^ ^44^, ^45^ ^]^ CD14‐positive monocytes exhibited high expression levels of *GZS1OOA12*, *CD14*, and *CVAN*. For the cell clusters predicted to be mature NK/T cells, we examined their gene expression profiles. Notably, they all greatly expressed the *CDKN1C* gene and presented low expression levels of the *C1Q* genes, which was consistent with the mature NK/T cell features instead of the original cell label “monocyte” (Figure 4i; Figures S7 and S8, Supporting Information).

We also identified a cluster of “poorly predicted” cells, which had a wide range of different ground‐truth labels, that UniMap classified as “monocytes” with low confidence. Through the analysis of their gene expression profiles, we also found that these cells presented expression patterns that were more similar to those of monocytes, regardless of their assigned ground‐truth labels (Figure 4d; Figure S9, Supporting Information). These findings further confirm the high reliability of the predictions yielded by UniMap.

### 2.5 UniMap Achieves Atlas‐Level Integration and High‐Resolution Cell Annotation

Given the high cost of large‐scale single‐cell sequencing, ideal reference atlases are often unavailable, especially for specialized biological contexts such as rare diseases.^[^ ^46^, ^47^ ^]^ This situation necessitates the integration of multiple “imperfect” datasets to construct comprehensive atlases for subsequent high‐precision annotation. To demonstrate UniMap's suitability for integrating existing partially overlapping atlases with varying annotation resolutions, we gathered two PBMC atlases from patients with severe myasthenia gravis (MG). The first dataset (D1)^[^ ^48^ ^]^ consists of 64649 cells across 49 cell types, primarily including B cells, myeloid cells, stromal cells, immature T cells, and mature T cells. The second dataset (D2)^[^ ^49^ ^]^ contains 53748 cells across 20 cell types, primarily including B cells, myeloid cells, and mature T cells. Notably, D1 encompasses most of the cell types present in D2 and offers higher annotation resolution. However, D2 includes three myeloid cell subtypes—neutrophils, neutrophil–myeloid progenitors, and megakaryocytes/platelets—that are not present in D1 (Figure S11, Supporting Information).

**Figure 5:**
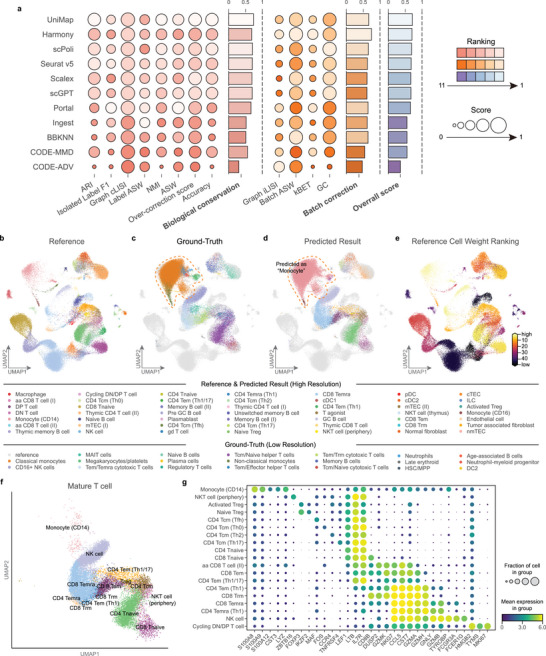
Effective integration and high‐resolution annotation of partially overlapping datasets with varying resolutions. a) Overview of all benchmark models by overall score based on PBMC MG datasets. Metrics are divided into biological conservation (red) and batch correction (orange). Overall scores are computed using the average of all individual metrics. All scores are normalized to a range of 0 to 1, with the higher values indicating better performance. b–e) UMAP plots of the integration results by UniMap, colored by reference cell types, query ground‐truth labels, predicted results and reference cell weight rankings. f) UMAP plot of the integration result by UniMap for cell populations with ground‐truth labels of mature T cells, colored by predicted labels. g) Dot plot showing expression of differentially expressed genes among the predicted subtypes of mature T cells, with the color and size representing normalized gene expression and the percentage of cells expressing a given gene, respectively.

To conduct a simple unbiased high‐resolution validation experiment, we partitioned Dl into query cells and reference cells at a ratio of 1:2. Our results demonstrate that UniMap achieves superior performance among all baseline models (Figure S10, Supporting Information). Subsequently, we manually annotated D2 using D1's annotation standards to obtain high‐resolution annotations for D2 (Table S2, Supporting Information). Then, we used D1 as the reference to annotate D2 to assess whether UniMap could accurately discern the correct cell subtype information for D2 (Figure S10, Supporting Information). The results (**Figure** **5**a) revealed that UniMap still remained the best‐performing model in terms of biological conservation, accurately annotating B cells into 5 subtypes and mature T cells into 19 subtypes. In contrast, while models like Harmony excelled in batch integration, they often lost biological information on different cell subtypes during batch correction, resulting in less satisfactory predictions (Figures S11 and S13, Supporting Information). Additionally, the cell type weight knowledge learned by UniMap closely aligned with the reference interfering cells, indicating that the model effectively mitigates the influence of interfering cells during the integration process (Figure 5b–d).

Subsequently, we examined the gene markers of the 19 cell subtypes originally categorized as mature T cells further verify the reliability of UniMap's predictions. As we expected, cells predicted to be monocytes showed a significant upregulation of S100A8 and S100A9 (Figure 5f). The monocytes exhibited significant upregulation for the *S100A8* and *S100A9*. NK T, activated T_regs_, naïve T_regs_, and various CD4‐positive T_cm_ cells expressed their marker genes (*ZBTB16*, *FOXP3*, *IKZF2*, *MAF*, *FOS*, *CCR4*, and *TNFRSF4*).^[^ ^48^ ^]^ All CD4‐positive and CD8‐positive cells were distinctly differentiated through their *IL7R* and *CD8B* expressions. Cells predicted as Temra demonstrated notable upregulation of the *GNLY* and *GZMB* genes.^[^ ^50^ ^]^ NK cells exhibited high expressions of *TYROBP*, *FCGR3A*, and *FCER1G*,^[^ ^51^, ^52^ ^]^ whereas cycling DN/DP T cells exhibited elevated expressions of *HMGB2*, *TYMS*, and *MKI67* ^[^ ^53^ ^]^ (Figure 5g). The results confirmed that UniMap retained most of the distinct information of cell subtypes during the integration process.

### 2.6 UniMap is Capable of Discovering New Cell Types to Complement the Existing Reference Atlas

After completing the aforementioned step, D1 and D2 were integrated into a shared low‐dimensional space, with D2 benefiting from higher‐resolution annotations. We subsequently used D2 to supplement D1 to construct a merged atlas. For the cell types in D2—neutrophils, neutrophil–myeloid progenitors, and megakaryocytes/platelets, we refined these cells using their ground‐truth labels. Because even after integration by UniMap, these cell types maintained distinct low‐dimensional features and formed separate clusters (Figure 5c).

**Figure 6:**
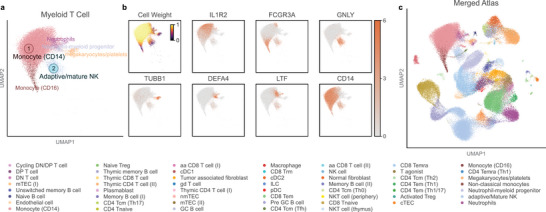
Construction of the comprehensive PBMC atlas for MG patients. a) UMAP plot of the integration result by UniMap for cell populations with predicted labels of monocytes from on PBMC MG dataset 1. The cells are colored by the detailed original ground‐truth labels, and aside from monocytes, Lovein clusters are used for annotation. b) UMAP plots showing the cell weights and expression of differentially expressed genes, with darker colors indicating higher gene expression. c) The new composite atlas including all labels from PBMC MG datasets 2, most labels from automatically annotated PBMC MG datasets 1, except for cells predicted to be monocytes, which are replaced with original ground‐truth labels and manually identified cell types.

However, upon forming a UMAP visualization, we observed distinct subclusters within the monocyte cluster, suggesting potentially undefined subtypes (Figure 5c). We employed the UniMap cell embeddings and applied the Leiden algorithm^[^ ^54^ ^]^ for unsupervised clustering, resulting in the identification of 2 relatively independent cell clusters (**Figure** **6**a). Cluster 1 exhibited high expressions of *IL1R2* and *RGS2*, which are known markers of CD14+ monocytes.^[^ ^55^, ^56^ ^]^ Cluster 2 displayed high expression levels for the *IFITM1*, *GNLY*, and *IL32* genes, which are known markers of adaptive NK cells^[^ ^57^ ^]^ (Figure 6b; Figure S14, Supporting Information). These refined annotations were added into the merged atlas, resulting in a more comprehensive PBMC atlas for MG patients (Figure 6c). This indicates that UniMap avoids over correction in its effort to remove batch effects and retains the intrinsic biological characteristics of different cells, thereby facilitating the identification of novel cell types and the construction of comprehensive atlases.

### 2.7 Extending UniMap to Integrate snRNA‐seq Data and scRNA‐seq Data

The integration of different single‐cell profiling methods, such as single‐cell RNA sequencing (scRNA‐seq) and single‐nucleus RNA sequencing (snRNA‐seq), introduces additional complexity in partial overlap datasets beyond that typically encountered in single‐modality analyses.^[^ ^58^ ^]^ To demonstrate UniMap's potential for integrating partial‐overlap datasets across different data types, we conducted an in‐depth experiment combining scRNA‐seq and snRNA‐seq datasets. The datasets were derived from human lung tissue,^[^ ^59^ ^]^ comprising snRNA‐seq (*n* = 63 745 cells) and scRNA‐seq (*n* = 129 079 cells), which captured distinct cellular populations (Figure S15, Supporting Information). Notably, snRNA‐seq exhibited superior detection of tissue‐resident cell types, including chondrocytes, submucosal glands, and alveolar type 1 and 2 cells (AT1, AT2), while scRNA‐seq primarily captured immune and circulation‐associated cells, such as erythrocytes, innate lymphoid cells (ILCs), alveolar macrophages, and monocytes.

To evaluate UniMap's cross‐modality performance on partial‐overlap datasets, we performed bidirectional annotation experiments: (1) using scRNA‐seq as the reference to annotate snRNA‐seq data, and (2) using snRNA‐seq as the reference to annotate scRNA‐seq data. UniMap demonstrated superior annotation accuracy in both scenarios, achieving accuracy rates of 84.8% and 81.4%, respectively. Comparative analysis with existing methods revealed that UniMap offered the most balanced performance, excelling in both biological conservation and batch correction, outperforming Harmony, Seurat v5, and BBKNN (Figures S16–S19, Supporting Information). These results underscore UniMap's versatility and robustness in integrating datasets from diverse modalities and scenarios.

### 2.8 UniMap Aligns Cell Atlases Across Multiple Species

Leveraging scRNA‐seq data to conduct cross‐species integration and annotation is essential for studying species diversity and interspecies variation.^[^ ^60^, ^61^ ^]^ It allows for the generation of annotation drafts for less‐studied species via data acquired from well‐researched species. Additionally, it enables detailed cross‐species comparisons, helping identify unique cell types within a specific species and cells with similar functionalities across different species. However, this task is rather challenging due to the inherent noise involved in interspecies comparisons, such as the differences between species‐specific gene expression patterns and the smaller number of genes shared between species.^[^ ^62^ ^]^ Furthermore, the cell types of different species do not align perfectly, resulting in the presence of interfering cells and types. Thus, an effective annotation tool should perform cell‐specific alignment and quantify prediction confidence to ensure reliable annotation results.

**Figure 7:**
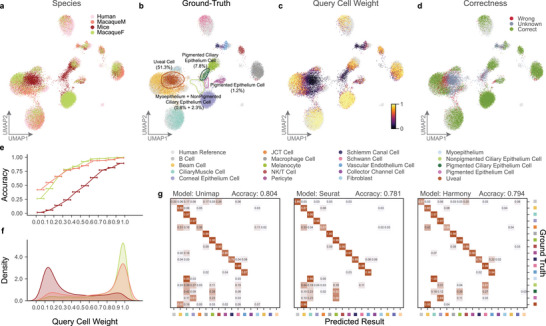
Cross‐Species Integration and Annotation with UniMap. a–d) UMAP plots of the integration results by UniMap, colored by species, ground‐truth labels, query cell weights, and correctness from Cross‐species dataset. e,f) Prediction accuracy and density for different cell weight intervals (each interval width is 0.1) across different query species. g) Confusion matrices comparing predicted labels by benchmark models (UniMap, Seurat v5, and Harmony) and the ground‐truth labels from the original publication.

We assessed the performance of UniMap via a cell atlas of the aqueous humor outflow pathway in the eyes,^[^ ^63^ ^]^ encompassing data from four species, where we manually aligned the names of identical cell types across different species (Table S3, Supporting Information). We compared the performance of various models in this task. Human scRNA‐seq data served as a reference for annotating the scRNA‐seq data of three other species: mice (*Mus musculus*), cynomolgus macaques (*Macaca fascicularis, MF)*, and rhesus macaques (*Macaca mulatta, MM*). Figure S20 (Supporting Information) details a comparative analysis of the performance of these models. Strong batch effects and cell type inconsistencies across species caused most models to fail in terms of achieving satisfactory integration results. Only UniMap and Harmony successfully aligned the data derived from different domains in a low‐dimensional space (**Figure** **7**a,b; Figure S21, Supporting Information).

As anticipated, the closer phylogenetic relationship of the *MM* and *MF* species to humans, along with the comprehensive coverage of almost all cell types in the reference atlas, facilitated precise mapping. The results predicted for the *MF* and *MM* query species were highly favorable, with accuracies of 94.2% and 89.2%, respectively, and higher cell weights (Figures S22 and S23, Supporting Information). In contrast, the mice species contained a substantial proportion of interfering cells (51.3% of the cells were uveal cells, 7.8% were pigmented ciliary epithelium cells, 2.3% were nonpigmented ciliary epithelium cells, 1.2% were pigmented epithelium cells, 0.8% were myoepithelium cells, accounting for 63.4% of the queried cell population) (Figure 7b; Figure S20, Supporting Information), which were absent in the reference atlas. UniMap was designed to recognize these “unpredictable” cells by outputting the cell weight for each cell, and classified those cells with cell weight below 0.25 as “unknown” (Figure 7c,d). Additionally, for query cells with labels that were present in the reference, UniMap still provided the most accurate predictions (Figure 7g).

We analyzed the distributions of the query cells across various cell weight intervals in different species. Query cells from MM and MF generally displayed higher cell weight levels, whereas those from mice exhibited a “bimodal distribution.” Additionally, the prediction results obtained for all the species were more accurate with increasing cell weight intervals (Figure 7e,f). In our analysis, low confidence cells strongly aligned with incorrectly predicted cells (Figure 7c,d; Figure S23, Supporting Information). To improve the integration process and annotation quality, we filtered out low‐weight cells that were beyond the predictive capacity of the model. For example, by removing cells with cell weight less than 0.25, the remaining cells yielded a prediction accuracy of 0.900, which was greater than the original value of 0.759 (Figure S24, Supporting Information).

To further assess the impact of prediction reliability on downstream analyses, we analyzed the expression of 13 interspecies‐conserved genes, previously identified as critical to the trabecular meshwork and uveoscleral outflow pathway,^[^ ^64^ ^]^ across four cell types—Schwann cells, melanocytes, ciliary muscle cells, and pericytes—under two conditions: with and without the filtering of low‐quality cells. For the MF and MM species, where the majority of cells exhibited high prediction confidence, the expression profiles showed minimal differences between the predicted results and the ground truth. However, for the Mice species, we observed that only after filtering out predictions with cell weights below 0.25 did the gene expression profiles align with those of the ground truth (Figure S25, Supporting Information), consistent with the aforementioned analysis. To further investigate, we analyzed 13 marker genes shared among 10 cell types across different species. The expression patterns of these genes were remarkably similar across species, including 6 previously reported markers,^[^ ^64^ ^]^ suggesting that the remaining genes may also be potential cross‐species conserved markers (Figures S26 and S27, Supporting Information).

## 3 Conclusion

To address the challenges posed by the “data‐drift problem” in real‐world single‐cell integration studies, we introduce UniMap, an end‐to‐end interpretable framework designed for atlas‐level data integration and high‐resolution cell annotation. UniMap excels in managing partially overlapping datasets and those with varying resolutions. Evaluated using 14 metrics related to biological conservation and batch correction, UniMap surpasses 10 state‐of‐the‐art models in several complex real‐world single‐cell integration tasks and provides more biologically meaningful predictions, including high‐precision cell subtype annotation, cell type alignment across single‐cell sequencing methods, construction of comprehensive atlases for specific biological contexts, discovery of novel cell types, annotation of cell states along developmental trajectories, and cross‐species cell type comparison studies.

Distinguished from traditional methods, UniMap achieves type‐level integration through a multi‐selective adversarial network architecture and dynamic cell weight mechanisms. Specifically, UniMap can accurately identify and exclude interfering cells from both the reference and the query datasets, reducing their impact on the integration process and avoiding over‐correction. Furthermore, it features an interpretable strategy that allows for direct observation of the similarities and differences between datasets, enhancing our understanding of the model's decision‐making process. UniMap also helps with improving downstream analyses by filtering out low‐quality predictions. These attributes make UniMap well‐suited for contemporary large‐scale and high‐quality research in life sciences and biomedicine. However, despite the utility of cell weights, UniMap currently faces a limitation in distinguishing whether cells with low weights are interfering cells or cells in transitional states. To address this challenge, we strongly recommend that researchers combine their understanding of the data context with UniMap's predictions and cell weights when making analytical decisions. This will ensure a more informed and nuanced interpretation of results.

In practical scenarios involving complex tasks such as resolving inconsistencies in label resolution, UniMap demonstrates strong capabilities in preserving biological information and maintaining cell subtype differences. However, manually aligning cell labels with different naming formats remains a time‐consuming but essential step due to the lack of standardized cell naming conventions. A potential solution is to combine UniMap with tools such as CellHint,^[^ ^65^ ^]^ which address naming issues, to achieve a more automated and universal single‐cell analysis framework.

On the other hand, UniMap currently uses shared highly variable genes among different datasets as input features and employs a simple MLP for feature extraction. Recent studies,^[^ ^66^ ^]^ however, have demonstrated that advanced single‐cell large language models (LLMs), such as scGPT and scFoundation,^[^ ^67^ ^]^ possess robust capabilities in efficiently extracting whole transcriptomic features and generating universal cell embeddings based on billion‐scale single‐cell pretraining datasets. Despite these advancements, some research suggests that these models may not outperform traditional models in integration, annotation, or other tasks, even with fine‐tuning.^[^ ^68^ ^]^ Potential improvements could involve using single‐cell LLMs for pre‐extracting cell features and integrating them with architectures similar to UniMap's to enhance prediction robustness.

We envision UniMap becoming a crucial tool for data integration and annotation given its improvements in terms of preserving biological signals and biological interpretability. The focus of our model on type‐specific alignment aims to offer researchers a fresh perspective, enhancing the reliability and interpretability of single‐cell analyses.

## 4 Experimental Section

### The Overview of UniMap

In this study, we propose UniMap, a model designed to isolate and extract pure biological signals from a diverse array of gene expression profiles, aiming to precise and interpretable transfer of knowledge from existing references to query cell populations. Inspired by several works^[^ ^69^ ^]^ in the field of transfer learning, UniMap employs three parts as its framework: a feature extraction module (E) for extracting low‐dimensional features of cells, a label prediction module (P) for predicting cell labels, and a type‐specific discrimination module (D) for evaluating the distribution alignment of low‐dimensional features across different domains at the cell type level. The following sections provide an in‐depth introduction to UniMap, followed by a comprehensive exploration of the three modules. The emphasis will be on the underlying data structures and algorithms.

### Glossary

This section outlines the key symbols and terms used in the discussion of UniMap, including quantitative parameters related to data dimensions and qualitative descriptions of the three main functional modules. Each symbol is defined to clarify its role and significance within the analysis framework (**Tables** **1** and **2**):

***m*** the number of reference domain cells (subscript ***r***)
***n*** the number of query domain cells (subscript ***q***)
***g*** the number of highly variable genes shared between different domains
***d*** the number of unique cell types in reference cells
***u*** the number of query domain cells with pseudo‐labels, a value that dynamically evolves throughout the training process
***c*** the dimensionality of the batch information embedding vectors
***z*** the dimensionality of the low‐dimensional features of cells
E the feature extraction module
P the label prediction module
D the type‐specific discrimination module

**Table 1:** Data related mathematical symbols.

Symbols related to the reference domain cells	
$X_{r}\in R^{m\times (g+c)}$	The inputs of reference cells, including gene expression matrices and batch information embedding vectors.
***Y***_**r**_ ∈ {0, 1}^*m* × *d*^	The one‐hot encoded labels of reference cells.
$E\left(X_{r}\right)\in R^{m\times z}$	The low‐dimensional features of reference cells.
$\;\hat{Y_{r}}=P\left(E(X_{r})\right)\in {\left[0,1\right]}^{m\times d}$	The encoded predicted labels of reference cells.
**Symbols related to the query domain cells**.	
$X_{q}\in R^{n\times (g+c)}$	The inputs of query cells.
***Y***_**q**,**self**_ ∈ {0, 1}^*u* × *d*^	The one‐hot encoded pseudo‐labels of query cells.
$E\left(X_{q}\right)\in R^{n\times z}$	The low‐dimensional features of query cells.
$\;\hat{Y_{q}}=P\left(E(X_{q})\right)\in {\left[0,1\right]}^{n\times d}$	The encoded predicted labels of query cells.
**Symbols related to both reference domain and query domain cells**.	
$X_{\mathrm{rq}}\in R^{\left(m+n)\times (g+c\right)}$	The inputs of all cells.
$E\left(X_{\mathrm{rq}}\right)\in R^{(m+n)\times z}$	The low‐dimensional features of all cells.
$P\left(E(X_{\mathrm{rq}})\right)\in {\left[0,1\right]}^{(m+n)\times d}$	The one‐hot encoded predicted labels of all cells.
***D***_**rq**_ ∈ {0, 1}^*m* + *n*^	The domain labels of all cells.
$D\left(E(X_{\mathrm{rq}})\right)\in {\left[0,1\right]}^{m+n}$	The predicted domain labels of all cells.

**Table 2:** Weights related mathematical symbols.

Symbols related to cell type.	
$w_{r}^{\prime }\in {\left[0,1\right]}^{d}$	Cell type weight generated from SoftMax result in the moduleP, prior to normalizing.
$w_{r}^{\mathrm{ct}}\in {\left[0,1\right]}^{d}$	Normalized cell type weight.
**Symbols related to cells**.	
$H_{\mathrm{rq}}^{\mathrm{cell}}\in {\left[0,H_{\max}\right]}^{m+n}$	Entropy value of all cells.
$R_{\mathrm{rq}}^{\mathrm{cell}}\in {\left[1,2\right]}^{m+n}$	Modified entropy value of all cells, corrected using the negative exponent method.
$w_{r}^{\mathrm{cell}}\in {\left[0,2\right]}^{m}$	Cell weight of reference cells.
$w_{q}^{\mathrm{cell}}\in {\left[1,2\right]}^{n}$	Cell weight of query cells.
$w_{\mathrm{rq}}^{\mathrm{cell}}\in {\left[0,1\right]}^{m+n}$	Normalized cell weight of all cells.

### Model Structure—Feature Extraction Module

In UniMap, the initial cell representations can be divided into two parts: the reference cells ***X***_**r**_ and the query cells ***X***_**q**_. Both are formed by concatenating gene expression and batch information. Gene expression data, comprising the expression levels of ***g*** highly variable genes per cell, forms the foundational segment of the initial cell representations. The batch information segment is currently represented as ***c***‐dimensional embeddings. Unlike traditional integer or one‐hot encodings, these are randomly initialized vectors that dynamically update during training, proving to be a superior approach for capturing diverse batch information.^[^ ^18^ ^]^ Subsequently, all cell representations ***X***_**rq**_ are encoded into batch‐invariant low‐dimensional features $E(X_{\mathrm{rq}})$ by a 3‐layer neural network (fully connected [*g* + *c*]‐ReLU‐DP‐ fully connected [512]‐ReLU‐DP‐ fully connected [128]) within the module E.

### Model Structure—Label Prediction Module

The label prediction module features a 2‐layer neural network (fully connected [128]‐ReLU‐DP‐ fully connected [*d*]) and a SoftMax layer. It predicts cell types using the batch‐invariant embeddings $E(X_{\mathrm{rq}})$ and generates two weights: a cell type weight ($w_{r}^{\mathrm{ct}}$) and a cell weight ($w_{\mathrm{rq}}^{\mathrm{cell}}$), both derived from SoftMax outputs. These weights are critical for the training phase and dynamically evolve during the training process. They influence the loss function calculation and guide the training, ultimately determining what the model learns from the data. Detailed methodologies for calculating these weights are provided below:

(1) $$w_{r}^{\prime }={\left\{\frac{1}{n}\sum_{i=1}^{n}p_{q}\left[i,j\right]\right\}}_{j=1}^{d}\;\in {\left[0,1\right]}^{d}$$

(2) $$w_{r}^{\mathrm{ct}}=\frac{w_{r}^{\prime }}{\max\left(w_{r}^{\prime }\right)}\;\in {\left[0,1\right]}^{d}$$

where ***p***_**q**_[***i***, ***j***] denotes the probability of the ***i***‐th query cell from query being predicted as cell type ***j*** after passing through the SoftMax Layer in the module P.

After calculating $w_{r}^{\mathrm{ct}}$, the module P processes the SoftMax results of each cell to derive the modified entropy $R_{\mathrm{rq}}^{\mathrm{cell}}$. This measure, an adaptation of the entropy value, ensures that lower entropy (indicating higher confidence) yields a greater value, representing the significance of each cell

(3) $$R_{\mathrm{rq}}^{\mathrm{cell}}={\left\{1+e^{\sum_{j=1}^{d}p_{\mathrm{rq}}\left[i,j\right]\log\left(p_{\mathrm{rq}}\left[i,j\right]\right)}\right\}}_{i=1}^{m\;+\;n}\;\in {\left[1,2\right]}^{m\;+\;n}$$

However, when calculating the cell weight, we aim to evaluate the confidence level of each cell and minimize the influence of interfering cell types within the references. Thus, we adopted the following formula to integrate cell type information into the computation of cell weight across different domains

(4) $$w_{q}^{\mathrm{cell}}=R_{q}^{\mathrm{cell}}={\left\{R_{\mathrm{rq}}^{\mathrm{cell}}\left[i\right]\right\}}_{i=m}^{m\;+\;n}\in {\left[1,2\right]}^{n}$$

(5) $$w_{r}^{\mathrm{cell}}={\left\{w_{r}^{\mathrm{ct}}\left[Y_{r}\left[i\right]\right]⊙R_{\mathrm{rq}}^{\mathrm{cell}}\left[i\right]\right\}}_{i=1}^{m}\in {\left[0,2\right]}^{m}$$

where $w_{r}^{\mathrm{ct}}\left[Y_{r}\left[i\right]\right]$ denotes the cell type weight corresponding to the ground‐truth label of the ***i***‐th cell. $R_{\mathrm{rq}}^{\mathrm{cell}}\left[i\right]$ represents the modified entropy of the ***i***‐th cell and ⊙ denotes the Hadamard product. For query domain cells, their cell weight $w_{q}^{\mathrm{cell}}$ equals their $R_{q}^{\mathrm{cell}}$. For reference cells, their cell weight is the product of their $R_{r}^{\mathrm{cell}}$ and the $w_{r}^{\mathrm{ct}}$ corresponding to their ground‐truth cell type. This approach aims to reduce the influence of interfering cell types in the references. Ultimately, we concatenate these weights to serve as the final composite cell weight $w_{\mathrm{rq}}^{\mathrm{cell}}$

(6) $$w_{\mathrm{rq}}^{\mathrm{cell}}=\frac{\mathrm{concat}\left(w_{r}^{\mathrm{cell}},w_{q}^{\mathrm{cell}}\right)}{2}\;\in {\left[0,1\right]}^{m\;+\;n}$$

### Model Structure—Type‐Specific Discrimination Module

The Type‐Specific Discrimination Module D includes one GRL and ***d*** discriminators with identical structures. They process the batch‐invariant embeddings $E(X_{\mathrm{rq}})$ to produce a discrimination result $\left(E(X_{\mathrm{rq}})\right)\in {\left[0,1\right]}^{m\;+\;n}$​, where 0 and 1 denote the reference and query domains, respectively. Controlled by $w_{r}^{\mathrm{ct}}$, each discriminator determines the domain of cells in a specific cell type. The GRL ensures that during gradient backpropagation, the parameter optimization directions in modules E and D are opposite. Thus, D enhances the ability to distinguish data sources, while the module E enhances the ability to confuse them. Ultimately, the parameters in themoduleE are influenced by the downstream loss functions from both P and D. This enables themoduleE to extract batch‐invariant low‐dimensional representations of cells with identical cell type labels across different sources.

### Model Training—Loss Function

In UniMap, parameter optimization uses four loss functions: $L_{r}$, $L_{q}$, $L_{\mathrm{trans}}$, and $L_{\mathrm{margin}}$. All of these are closely related to $w_{r}^{\mathrm{ct}}$ and $w_{\mathrm{rq}}^{\mathrm{cell}}$. Among these, $\;L_{r}$ and $L_{q}$ are defined as classification losses as follows

(7) $$L_{r}=w_{r}^{\mathrm{cell}}\;\cdot l_{\mathrm{fl}}\left(Y_{r},\hat{Y_{r}}\right)$$

(8) $$L_{q}=w_{q}^{\mathrm{cell}}\;\cdot l_{\mathrm{fl}}\left(Y_{q,\mathrm{self}},{\hat{Y}}_{q}\right)$$

where $w_{r}^{\mathrm{cell}}$ and $w_{q}^{\mathrm{cell}}$ denote the cell weights for the reference domain and query domain, respectively. $l_{\mathrm{fl}}\left(Y,\hat{Y}\right)$ represents the focal loss^[^ ^70^ ^]^ between ground‐truth labels and predicted labels. Due to the lack of true labels in query domain cells, we adopt a pseudo‐label strategy to reduce prediction uncertainty. This strategy involves iteratively assigning pseudo‐labels to query domain cells with maximum SoftMax probability (Predicted Probability) > *threshold* (default value of 0.9). We then retrain UniMap using ground‐truth labels from the reference domain and pseudo‐labels from the query domain.

$L_{\mathrm{trans}}$ is a loss function that measures the mixing of low‐dimensional cell representations from different domains per cell type, indicating the degree of batch information removal. The calculation formula is as follows

(9) $$L_{\mathrm{trans}}=\sum_{j=1}^{d}w_{r}^{\mathrm{ct}}\left[j\right]\;\cdot w_{\mathrm{rq}}^{\mathrm{cell}}⊙p_{\mathrm{rq}}\left[:,j\right]\cdot l_{\mathrm{ce}}\left(D_{j}E\left(X_{\mathrm{rq}}\right),D_{\mathrm{rq}}\right)$$

This loss function comprises three parts, with the most significant part being $l_{\mathrm{ce}}\left(D_{j}E\left(X_{\mathrm{rq}}\right),D_{\mathrm{rq}}\right)$. It measures the cross entropy between the judgment result $D_{j}E\left(X_{\mathrm{rq}}\right)$ from discriminator $D_{j}$ and the real domain labels ***D***_**rq**_ across all cells. To ensure that $D_{j}$ specifically handles cells categorized as ***j*** or predicted as ***j***, we include the coefficient ***p***_**rq**_[:, ***j***] representing the probability of cells being predicted as ***j***. The term $w_{r}^{\mathrm{ct}}\left[j\right]\cdot w_{\mathrm{rq}}^{\mathrm{cell}}$​ ensures distinct weights for different cells and cell types in the loss calculation. This enables UniMap to prioritize weights for reference cells and valuable cell types when mapping query cells to the reference atlas.

$L_{\mathrm{margin}}$ is defined as the sum of weighted logarithmic ratios of predicted probabilities for each cell type, adjusted by the true cell type probability. This loss penalizes incorrect predictions by comparing them to the correct cell type, enhancing model differentiation between cell types. The calculation formula is as follows

(10) $$\begin{array}{ccc} L_{\mathrm{margin}} & = & \sum_{i=1}^{m}\sum_{j=1}^{d}\left(w_{r}^{\mathrm{cell}}\cdot \frac{p_{r}\left[i,j\right]}{1-p_{r}\left[i,Y_{r}\left[i\right]\right]}\cdot \ln\left(\frac{p_{r}\left[i,j\right]}{1-p_{r}\left[i,Y_{r}\left[i\right]\right]}\right)\right.\\ & & \left.\cdot \frac{1_{j\;\neq \;Y_{r}\left[i\right]}}{\ln\left(d-1\right)}\right) \end{array}$$

where ***p***_**r**_[***i***, ***j***] and ***p***_**r**_[***i***, ***Y***_**r**_[***i***]] denote the probabilities of reference cells being predicted as ***j*** and their ground‐truth labels by the module P. $1_{j\neq Y_{r}\left[i\right]}$ equals 1 if ***j*** is not the ground‐truth label of ***i***‐th cell, otherwise it equals 0.

Therefore, the overall optimization objective for UniMap can be formulated as

(11) $$L\;\left(\theta_{E},\theta_{D},\theta_{P}\right)=L_{r}+\alpha \cdot L_{q}-\beta \cdot L_{\mathrm{trans}}+\gamma \cdot L_{\mathrm{margin}}$$

where $\theta_{E}$ represents the parameters of the module E, $\theta_{D}$ represents the parameters of the module D, and $\theta_{P}$ represents the parameters of the module P. The coefficients ***α***, ***β***, and ***γ*** denote the weight proportions between different loss functions, with default values of 0.5, 0.5, and 1 respectively. Therefore, the entire optimization process becomes a process of “maximizing and minimizing,” where continuous training strives to find an optimal set of parameters that satisfy

(13) $$\left(\theta_{E},\theta_{P}\right)=\underset{{\hat{\theta }}_{E},{\hat{\theta }}_{P}}{\arg\;\min}L\left(\theta_{E},\theta_{D},\theta_{P}\right)$$

(13) $$\left({\hat{\theta }}_{D}\right)=\underset{{\hat{\theta }}_{D}}{\arg\;\max}L\left(\theta_{E},\theta_{D},\theta_{P}\right)$$

### Model Training—Optimization and Other Hyperparameters

We used the Adam^[^ ^71^ ^]^ optimizer with a weight decay of 5e‐4 and set the initial learning rate to 1e‐4. Additionally, we employed a learning rate scheduling strategy to iteratively optimize the model: the learning rate decreases to 60% of its current value every 5 epochs during training (**Table** **3**). The maximum number of training epochs varies based on the task, typically set to 50, and training halts early if there's no improvement for 10 consecutive epochs. Furthermore, we systematically documented additional hyperparameters used in model training in **Table** **4**.

**Table 3:** Maximum training epochs for different tasks.

Task	Maximum training epoch
PBMC CVID	50
PBMC COVID‐19	3
PBMC MG	15
Cross‐Species	50
BMO	50
Lung (scRNA‐seq as query)	20
Lung (snRNA‐seq as query)	50

**Table 4:** Hyperparameters Employed in Model Training.

Hyperparameter	Search Range	Optimal Value
*threshold*	[0.85, 0.90, 0.95]	0.90
α	[0.1, 0.5, 1.0]	0.5
β	[0.1, 0.5, 1.0]	0.5
γ	[0.1, 0.5, 1.0]	1.0

### Evaluation Metrics

To quantify the quality of UniMap and other benchmark models in the integration and annotation tasks, we have used batch correction matrices and biological conservation metrics. For the purpose of comparison, all metrics have been normalized to a range of 0 to 1, where larger values indicate superior batch integration performance or better preservation of biological information. Most of the metrics were calculated using the “scib” and “sklearn” package in Python, except for the Over‐Correction Score and the adjusted Shannon diversity index. Detailed information can be found in the description below

**ARI**. The adjusted Rand index (ARI) quantifies the level of agreement between two clustering results.

**Isolated label F1**. The isolated Label F1 evaluates how well isolated labels are distinguished from other labels in data‐driven clustering by using the F1 score to compare clustering results with ground‐truth labels.

**Graph cLISI**. The original cLISI quantifies the effective number of cell types in a neighborhood, where a higher value reflects a mix of different cell populations. The graph cLISI builds on this by enabling computations on graphs.

**ASW**. The average silhouette width is a metric designed to assess the clustering effectiveness, evaluating the cohesion of each data point within its assigned cluster and the separation from other clusters. It ranges from −1 to 1, where a score close to 1 indicates that data points match well within their clusters and have good separation from other clusters. A score close to 0 suggests that data points are near the boundary between clusters, and a score close to −1 indicates a higher likelihood of data points being assigned to the wrong cluster.

**Label ASW**. The label ASW evaluates ASW with respect to cell type labels.

**NMI**. The normalized mutual information (NMI) is a metric designed to assess the similarity between two clustering results.

**Over‐Correction Score**. The over‐correction score, introduced in Scalex, is used to evaluate the extent of overcorrection in the presence of batch effects by computing the percentage of cells with inconsistent cell types in each cell's neighborhood.

**ACC**. The accuracy refers to the percentage of correctly predicted cells out of the total number of queried cells.

**Graph iLISI**. The original iLISI measures the effective number of datasets within a neighborhood, while Graph iLISI extends this concept to enable calculations on graphs.

**Batch ASW**. The batch ASW evaluates ASW with respect to cell batches.

**kBET**. The k‐nearest neighbor batch effect test assesses the bias of a batch variable within the kNN graph by calculating the average rejection rate from Chi‐squared tests comparing local and global batch label distributions.

**GC**. The graph connectivity represents the average connectivity of the subgraph for all cell type labels, quantifies the connectivity per cell type label.

**Adjusted Shannon diversity index**. The Shannon diversity index^[^ ^72^ ^]^ is a metric employed to quantify the diversity within a biological system. In this study, we have adapted it to characterize the diversity of true labels among cells predicted to belong to the same cell type. This index ranges from 0 to 1, where a value closer to 1 signifies greater similarity in the proportions of cells from each cell type in the predicted results compared to the ground‐truth labels, and lower values indicate greater divergence. Its calculation formula is as follows

(14) $$\begin{array}{ccc} & & \mathrm{Adjusted}\;\mathrm{Shannon}\;\mathrm{diversity}\;\mathrm{index}\\ & & \;=\frac{\log\left(d\right)-\frac{1}{k}\sum_{j=1}^{k}\left(-\sum_{l=1}^{N_{j}}o_{l}\left({\hat{c}}_{j}\right)\log\left(o_{l}\left({\hat{c}}_{j}\right)\right)\right)}{\log\left(d\right)}\; \end{array}$$

where ***k*** is the total number of unique predicted cell types, ***N***_**j**_​ is the number of unique ground‐truth cell types within cells predicted as cell type **j**, and $o_{l}\left({\hat{c}}_{j}\right)$ represents the proportion of cells with ground‐truth label **l** within cells predicted as cell type **j**.

### Data Preprocessing

For the raw data, we followed the standard preprocessing workflow recommended by the Scanpy pipeline.^[^ ^33^ ^]^ Initially, we applied quality control by filtering out cells expressing fewer than 200 genes and genes expressed in fewer than 3 cells. Subsequently, we performed log‐normalization, scaling the gene counts of each cell to 1e6 counts per million (CPM), followed by a logarithmic transformation using the function log(1 + *x*) to ensure comparability of counts across cells. To identify highly variable genes within each batch of data, we selected the top ***G*** genes based on their dispersion, while controlling for means. Throughout most analyses, including within‐batch studies, we set ***G *** =  1200. However, in cross‐species analyses where the overlap of genes between species was limited, we increased ***G *** =  1400. Finally, we used the union of shared highly variable genes, totaling ***g*** genes, across different batches as input features for subsequent analyses.

### UMAP Visualization

For the raw data visualization, we first performed PCA on highly variable genes to obtain PCA features, which were then used as embeddings for UMAP projection. For other comparative analyses, UMAP was directly applied to the embeddings generated by each model.

### Statistical Analysis

For selecting highly variable genes, all single‐cell datasets were processed using the “scanpy.pp.highly_variable_genes” function from the Scanpy package, employing the “Seurat” method for variance stabilization. The top variable genes were selected based on the “n_top_genes” parameter to serve as input features. For differential expression analysis across all experiments, we used the “scanpy.tl.rank_genes_groups” function, applying the *t*‐test method. This statistical approach computed gene‐wise *t*‐statistics between groups, with *p*‐values adjusted for multiple testing using the Benjamini–Hochberg procedure. Genes with adjusted *p*‐values < 0.05 were considered significantly differentially expressed. The differential expression analysis yielded log2 fold changes and adjusted *p*‐values for each gene, facilitating the identification of statistically significant differentially expressed genes between groups.

## Conflict of Interest

The authors declare no conflict of interest.

## Author Contributions

H.H. and Y.G. should be regarded as joint first authors. H.H. and Y.G. conceived and designed the project. H.H. and Y.G. designed and implemented the computational framework and conducted benchmarks and case studies under the guidance of B.Y. C.H., and J.C. F.G. performed manual annotation of the single‐cell data. H.Y., Z.Z., H.Z. and F.Y. provided critical feedback during the study. Q.Y. and J.W. helped revise the manuscript.

## Supporting information



Supporting Information

Supplemental Table 1

## Data Availability

All data used in this work are publicly available. For PBMC CVID datasets was downloaded from CZ CELLxGENE Discover (https://cellxgene.cziscience.com/e/1e5bd3b8‐6a0e‐4959‐8d69‐cafed30fe814.cxg/); for BM datasets was downloaded from CZ CELLxGENE Discover (https://cellxgene.cziscience.com/collections/59cd85c5‐3b22‐4035‐b628‐2a20810ad54b); for PBMC COVID‐19 reference dataset from CZ CELLxGENE Discover (https://cellxgene.cziscience.com/e/c7775e88‐49bf‐4ba2‐a03b‐93f00447c958.cxg/); for PBMC COVID‐19 query dataset from CZ CELLxGENE Discover (https://cellxgene.cziscience.com/e/fa8605cf‐f27e‐44af‐ac2a‐476bee4410d3.cxg/); for PBMC MG dataset 1 from Single Cell Portal (https://singlecell.broadinstitute.org/single_cell/study/SCP1532/thymoma‐and‐pbmc‐of‐myasthenia‐gravis‐patients); for PBMC MG dataset 2 from GEO (https://www.ncbi.nlm.nih.gov/geo/query/acc.cgi?acc=GSE222427); for Lung datasets from HLCA (https://5locationslung.cellgeni.sanger.ac.uk/cellxgene.html); for Cross‐Species datasets from Single Cell Portal (https://singlecell.broadinstitute.org/single_cell/study/SCP780/cell‐atlas‐of‐aqueous‐humor‐outflow‐pathways‐in‐eyes‐of‐humans‐and‐four‐model‐species‐provides‐insights‐into‐glaucoma‐pathogenesis). Additionally, the codes for reproducing the results are available at https://github.com/Huahuatii/Reproducing–UniMap or https://zenodo.org/records/13627800.
