# Supplementary material for: UniMap: Type‐Level Integration Enhances Biological Preservation and Interpretability in Single‐Cell Annotation
Source: Adv Sci (Weinh). 2025 Feb 27;12(16):2410790. doi: 10.1002/advs.202410790 (PMC12021081; doi:10.1002/advs.202410790)
Supplement: Supplementary file 1 — Supporting Information [file ADVS-12-2410790-s001.pdf]

ADVANCED SCIENCE
Open Access

Supporting Information

for *Adv. Sci.*, DOI 10.1002/advs.202410790

UniMap: Type-Level Integration Enhances Biological Preservation and Interpretability in Single-Cell Annotation

Haitao Hu, Yue Guo, Fujing Ge, Hao Yin, Hao Zhang, Zhesheng Zhou, Fangjie Yan, Qing Ye, Jialu Wu, Ji Cao, Chang-Yu Hsieh* and Bo Yang**

UniMap: Type-Level Integration Enhances Biological Preservation and Interpretability in Single-Cell Annotation

Haitao Hu[1,7,#], *Yue Guo*[1,#], *Fujing Ge, Hao Yin*[1,7], *Hao Zhang*[1,7], *Zhesheng Zhou*[1], *Fangjie Yan*[1], *Qing Ye*[5], *Jialu Wu*[5], *Ji Cao*[1,2,3,6,*], *Chang-Yu Hsieh*[2,5,*], *Bo Yang*[1,2,3,4,*]

The first two authors should be regarded as Joint First Authors

Haitao Hu, Yue Guo, *Fujing Ge,* Hao Yin, Hao Zhang, Zhesheng Zhou, Fangjie Yan, Ji Cao, Bo Yang
1, Institute of Pharmacology and Toxicology, Zhejiang Province Key Laboratory of Anti-Cancer Drug Research, College of Pharmaceutical Sciences, Zhejiang University, Hangzhou 310058, China.
E-mail: Yang924@zju.edu.cn, kimhsieh@zju.edu.cn, caoji88@zju.edu.cn

Ji Cao, Chang-Yu Hsieh, Bo Yang
2, The Innovation Institute for Artificial Intelligence in Medicine, Zhejiang University, Hangzhou 310018, China.

Ji Cao, Bo Yang
3, Engineering Research Center of Innovative Anticancer Drugs, Ministry of Education, Hangzhou 310000, China.

Bo Yang
4, School of Medicine, Hangzhou City University, Hangzhou 310015, China.

Qing Ye, Jialu Wu, Chang-Yu Hsieh
5, College of Pharmaceutical Sciences, Zhejiang University, Hangzhou 310058, Zhejiang, P. R. China.

Ji Cao
6, Center for Medical Research and Innovation in Digestive System Tumors, Ministry of Education, Hangzhou 310020, China.

Haitao Hu, Hao Yin, Hao Zhang
7, Polytechnic Institute of Zhejiang University, Zhejiang University, Hangzhou 310015, China.

Supporting Information

1 Comparisons of Computational Costs of Benchmark Models on Four Datasets 1

2 Comparisons of integration and annotation results of benchmark models on PBMC CVID dataset 2

3 Comparisons of integration results of benchmark models on PBMC COVID-19 datasets....... 3

4 Comparisons of integration results of benchmark models on PBMC COVID-19 datasets (manual annotated labels) 4

5 Comparisons of annotation results of benchmark models on PBMC COVID-19 datasets 5

6 Comparisons of the adjusted Shannon diversity index of benchmark models on PBMC COVID-19 datasets 6

7 Canonical marker genes of predicted subtype results on the PBMC COVID-19 datasets 7

8 Reference and predicted results of canonical marker genes for monocyte cells and UMAP embeddings on the PBMC COVID-19 datasets 8

9 Gene expression differences between 'poorly predicted' and 'correctly classified' cells on the PBMC COVID-19 datasets 9

10 Comparisons of integration results of benchmark models on MG-part datasets..................... 10

11 Comparisons of integration results of benchmark models on PBMC MG datasets 11

12 Comparisons of integration results of benchmark models on PBMC MG datasets (manual annotated labels) 12

13 Comparisons of annotation results of benchmark models on PBMC MG datasets................ 13

14 Canonical marker genes of monocyte population on the PBMC MG dataset 1 14

15 Distribution of Cell Types in the Lung dataset 15

16 Predictive Performance and Benchmarking Analysis of UniMap Applied to the lung dataset 16

17 Comparisons of integration and annotation performance by quantification metrics............... 17

18 Comparisons of integration results of benchmark models on Lung datasets (using snRNA-seq as reference to annotate scRNA-seq) 18

19 Comparisons of integration results of benchmark models on Lung datasets (using scRNA-seq as reference to annotate snRNA-seq) 19

20 Composition of the Cross-species dataset and comparisons of integration performance by quantification metrics.. 20

21 Comparisons of integration results of benchmark models on Cross-Species dataset............. 21

22 Comparisons of annotation results of benchmark models on Cross-Species dataset 22-24

23 Comparisons of cell weights across different species 25

24 Impact of setting different cell weight thresholds on predicted results 26

25 Comparison of gene expression patterns between ground-truth and predicted results 27

26 Marker genes identified across different species 28

27 Comparison of gene expression patterns between different species 29

T1 Marker Genes for Manual Cell Type Annotation in the PBMC-COVID19 Dataset 30

T2 Marker Genes for Manual Cell Type Annotation in the MG Dataset 31

T3 Unified Naming Rules for Cross-Species Datasets 32

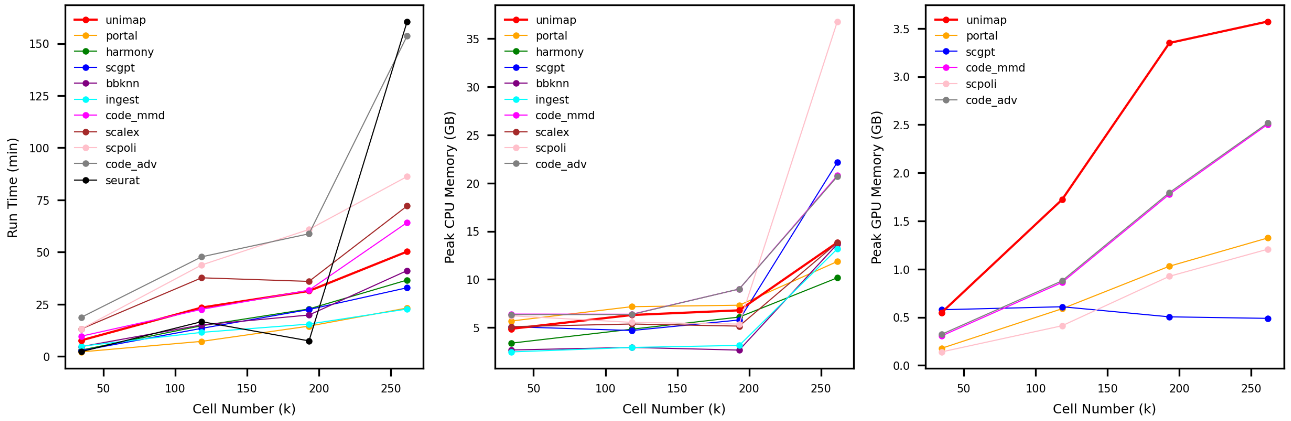

Figure S1. Comparisons of Computational Costs of Benchmark Models on Four Datasets. The computational time, peak CPU memory, and peak GPU memory (from left to right) were recorded using datasets with total cell counts (combined reference and query cells) of 34,600, 118,397, 193,108, and 261,386. For clarity, we did not display the CPU memory usage of Seurat, as it required 26.42 GB on the 118,397-cell dataset. The GPU memory consumption only shows the models that require GPU usage. Similarly, models that did not utilize GPU are not shown in the Peak GPU memory plot.

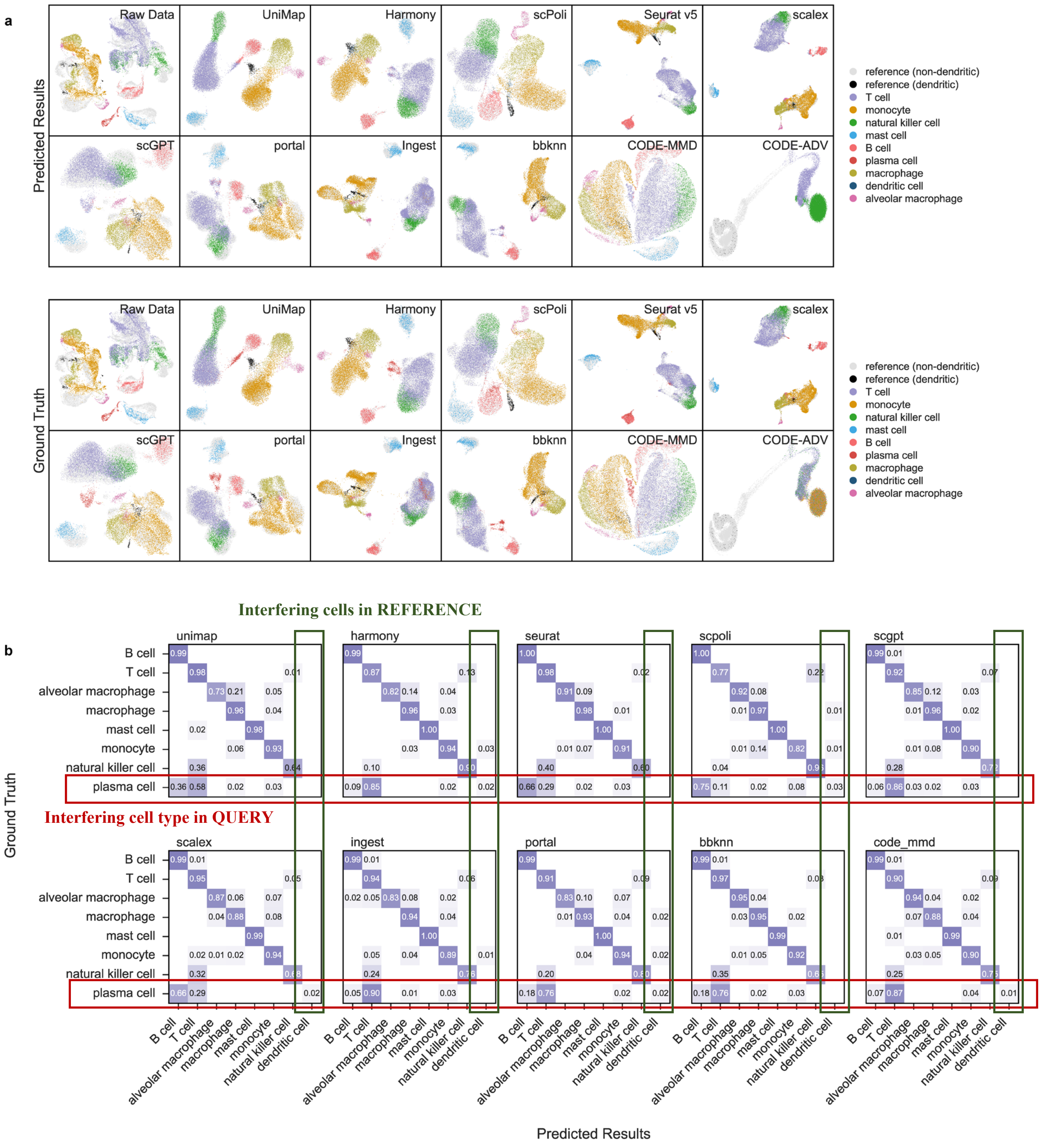

Figure S2. Comparisons of integration and annotation results of benchmark models on PBMC CVID dataset. a) UMAP plots showing the raw data and the integration results of all benchmark models (UniMap, Harmony, scPoli, Seurat v5, Scalex, scGPT, Portal, Ingest, BBKNN, CODE-MMD, and CODE-ADV) on the PBMC CVID dataset, colored by predicted labels and ground-truth labels. b) Confusion matrix showing the comparison of benchmark models in terms of predicted results. Interfering cell type in query (plasma cell) and reference (dendritic cell) are highlighted with red and green boxes, respectively.

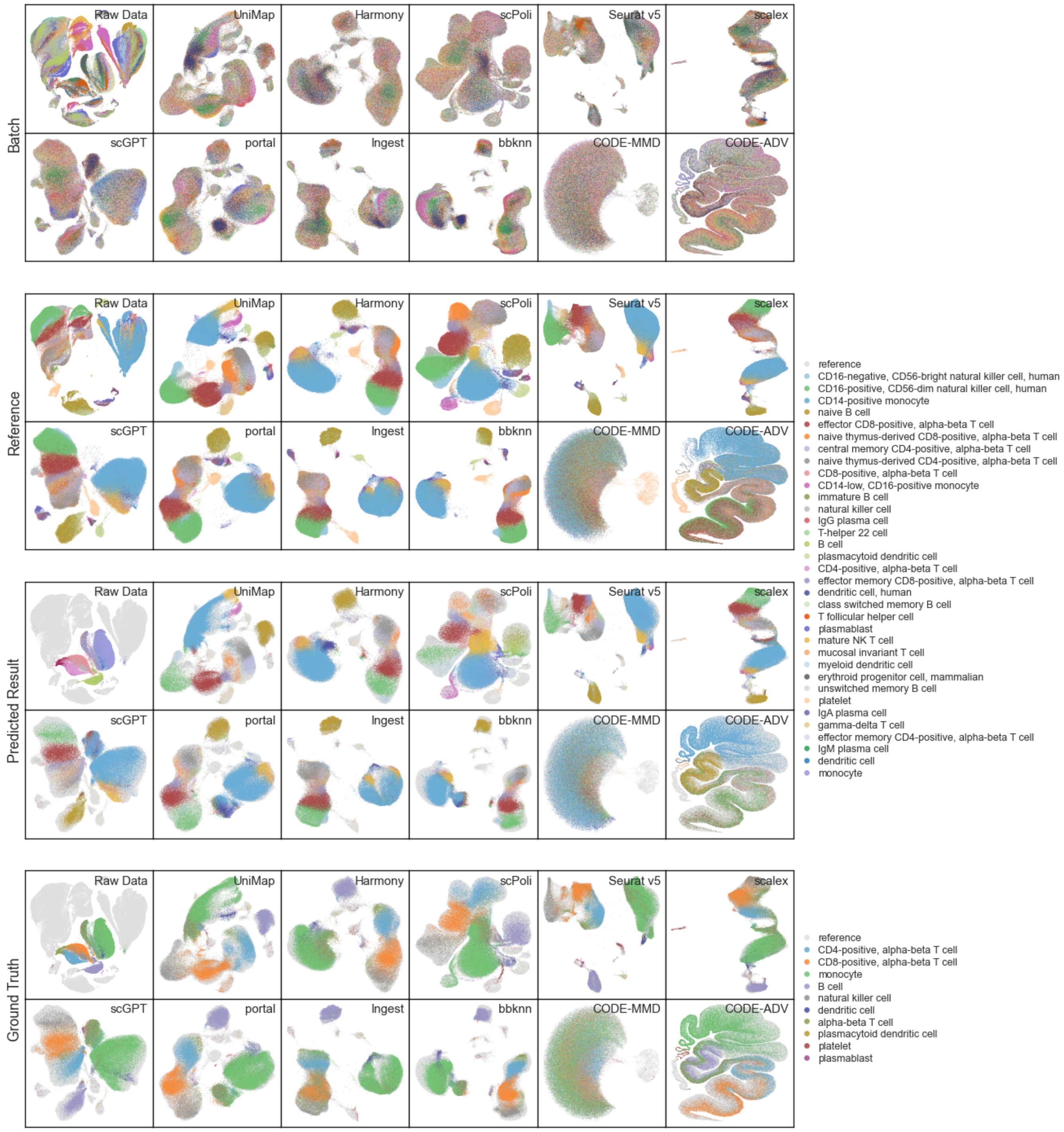

Figure S3. Comparisons of integration results of benchmark models on PBMC COVID-19 datasets. UMAP plots showing the raw data and the integration results of all benchmark models on the PBMC COVID-19 datasets, colored by batches, reference cell types, predicted results and ground-truth labels.

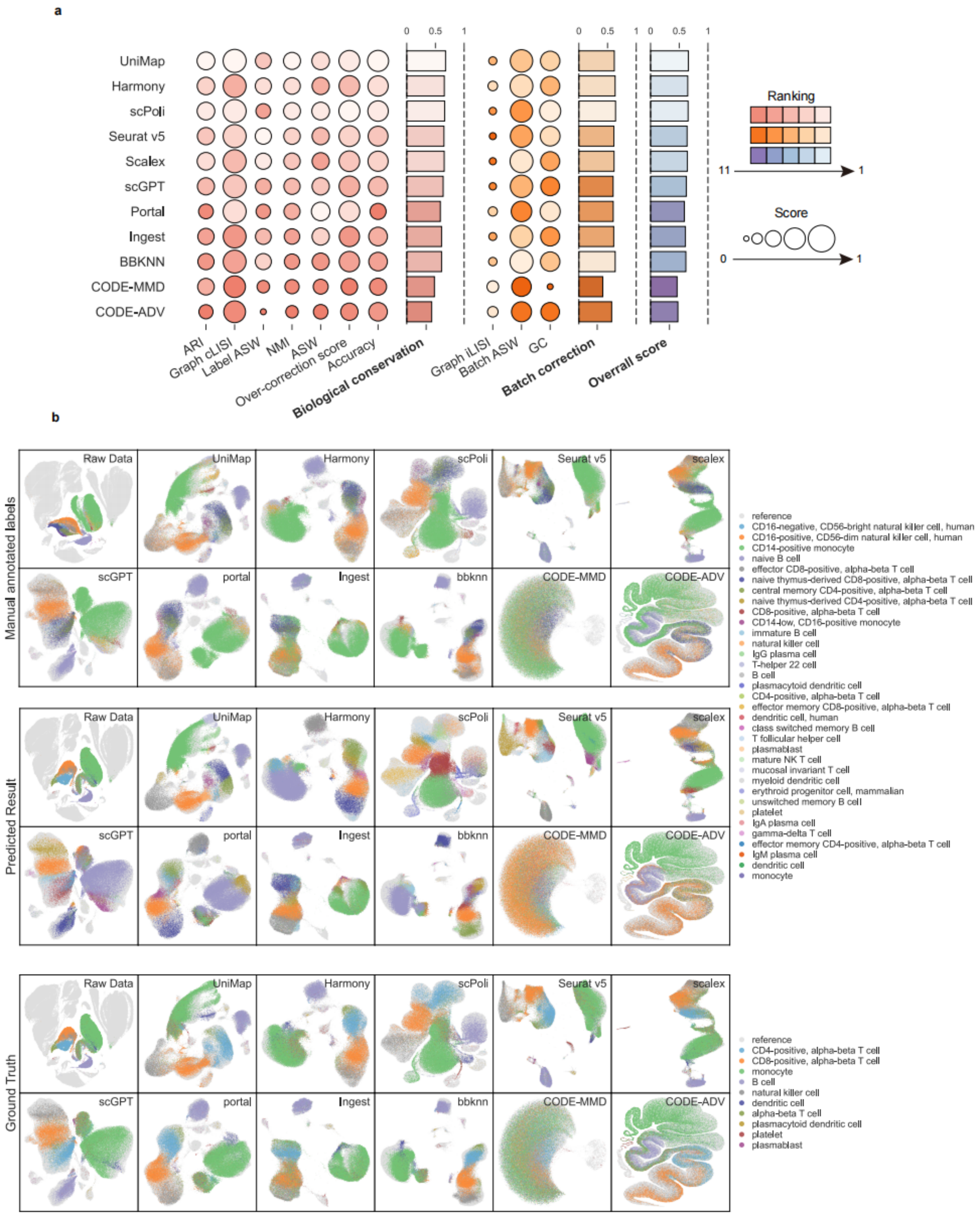

Figure S4. Comparisons of integration results of benchmark models on PBMC COVID-19 datasets (manual annotated labels). a) Overview of all benchmark models by overall score (purple) based on the MG-part datasets. b) UMAP plots showing the raw data and the integration results of all benchmark models on the PBMC COVID-19 datasets, colored by manual annotated labels, predicted results, and ground truth labels.

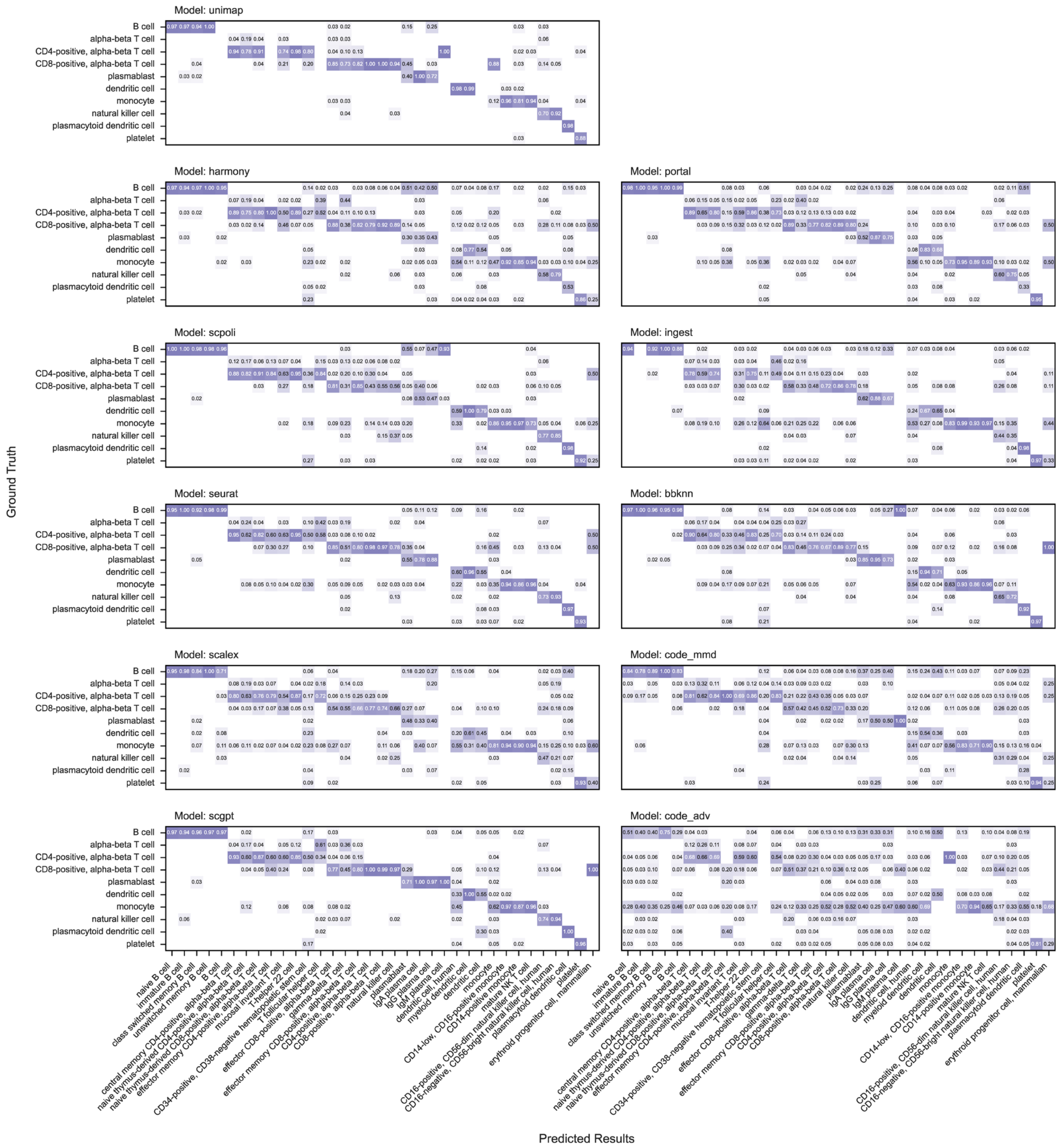

Figure S5. Comparisons of annotation results of benchmark models on PBMC COVID-19 datasets. Confusion matrix showing the comparison of benchmark models in terms of predicted results on the PBMC COVID-19 datasets.

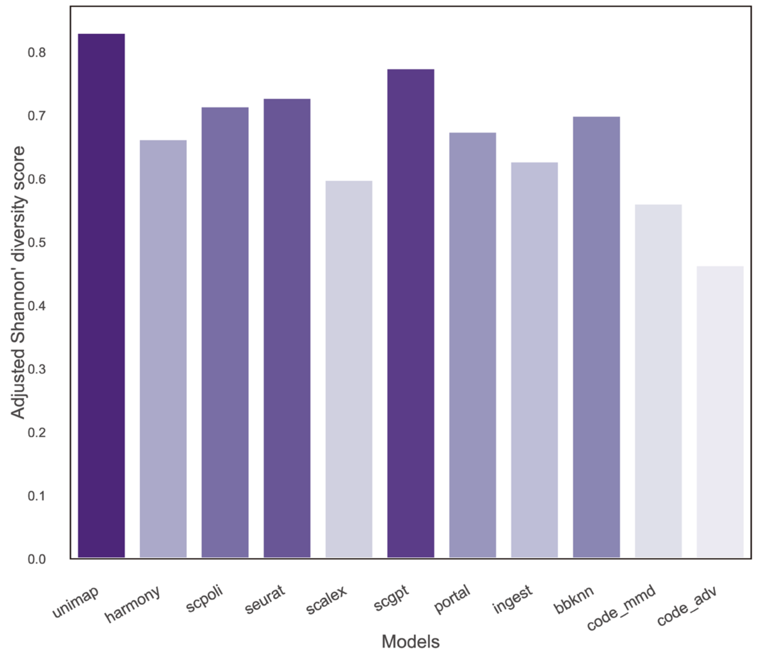

Figure S6. Comparisons of the adjusted Shannon diversity index of benchmark models on PBMC COVID-19 datasets. Bar plot showing the comparison of benchmark models in terms of the adjusted Shannon diversity index on the PBMC COVID-19 datasets, with darker colors indicating better rankings.

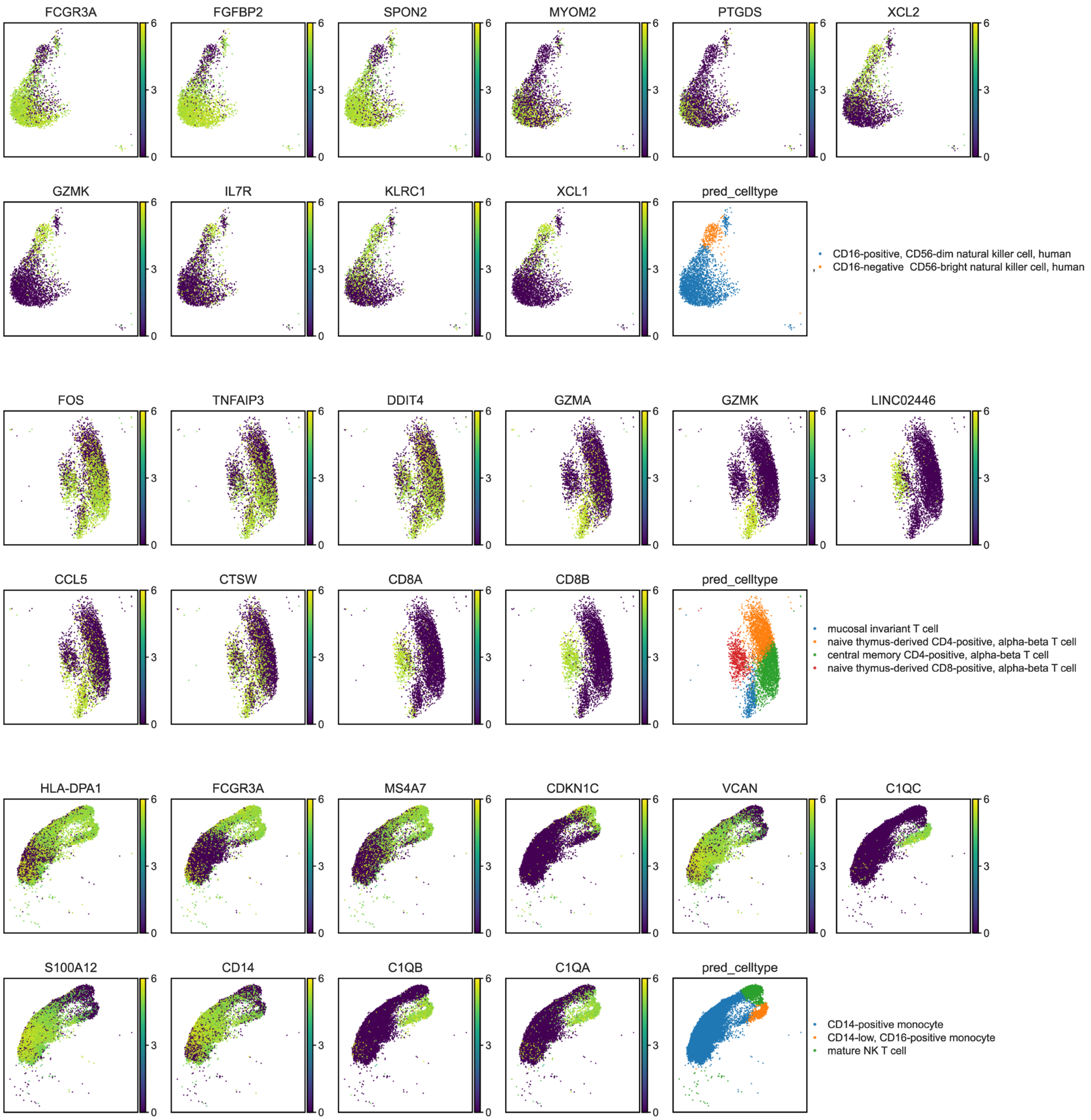

Figure S7. Canonical marker genes of predicted subtype results on the PBMC COVID-19 datasets. UMAP plots showing expression of differentially expressed genes and predicted subtype results by UniMap for different cell types (NK cells, CD4 αβ+ T cells, and monocytes) on the PBMC COVID-19 datasets.

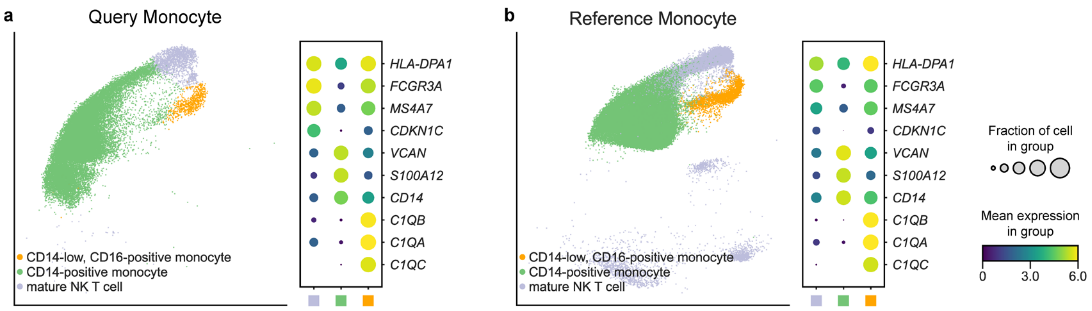

Figure S8. Reference and predicted results of canonical marker genes for monocyte cells and UMAP embeddings on the PBMC COVID-19 datasets. a, b) Left: UMAP plots of the integration results by UniMap for query and reference monocytes on the PBMC COVID-19 datasets, colored by predicted result and ground-truth labels. Right: Dot plot showing expression of differentially expressed genes among monocytes.

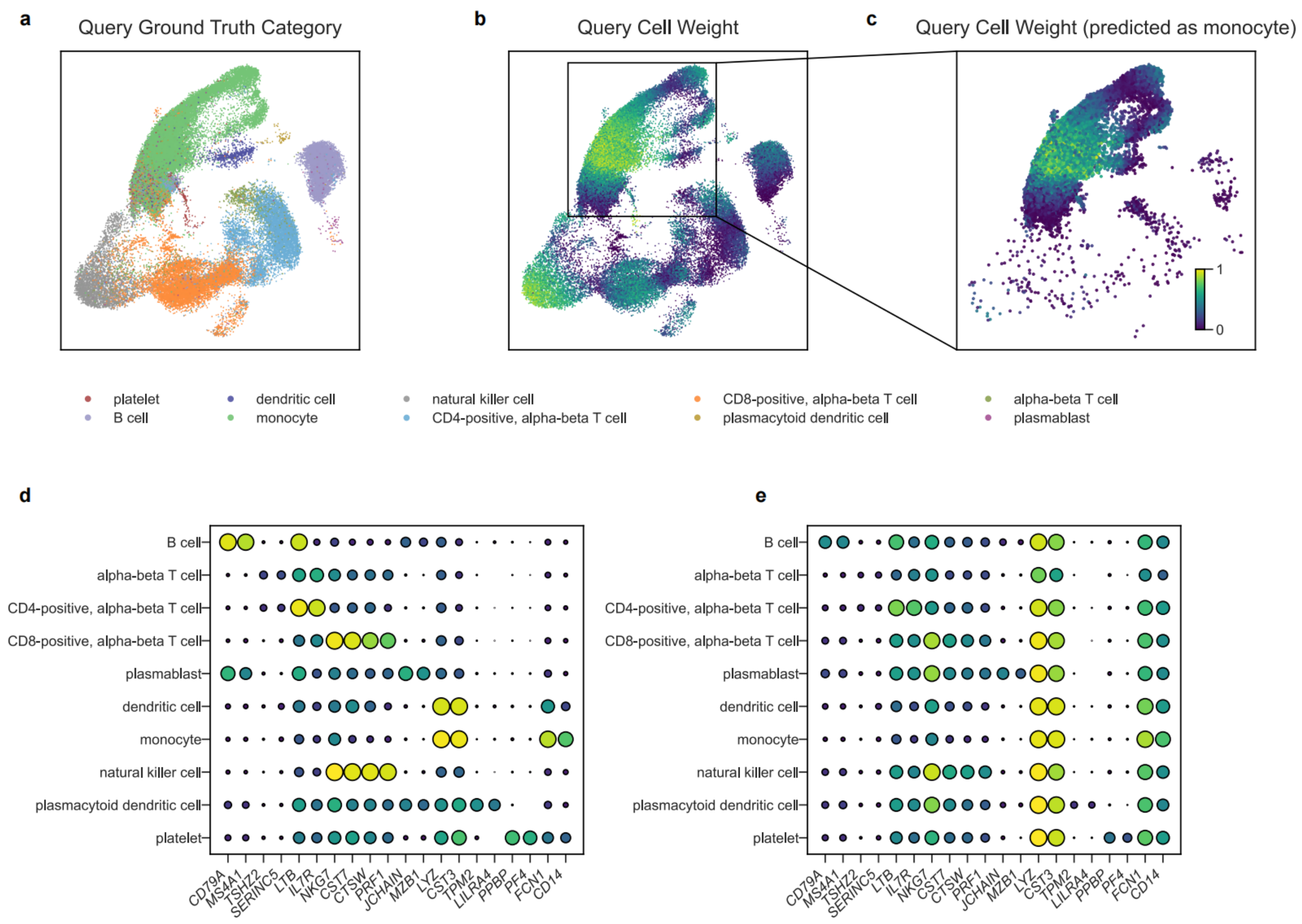

Figure S9. Gene expression differences between 'poorly predicted' and 'correctly classified' cells on the PBMC COVID-19 datasets. a) UMAP plot of the integration results by UniMap on the PBMC COVID-19 datasets, colored by ground-truth labels. b) UMAP plot of the integration results by UniMap for query cells, colored by query cell weights. c) UMAP plot of the integration results by UniMap for query monocytes, colored by query cell weights. d, e) Dot plots showing expression of differentially expressed genes in correctly predicted and poorly predicted cells, with the color and size representing normalized gene expression and the percentage of cells expressing a given gene, respectively. Correctly predicted cells are those whose predicted results match the ground-truth labels, while poorly predicted cells are query cells from various cell types that are predicted as monocytes, highlighted with brown circles in the plot.

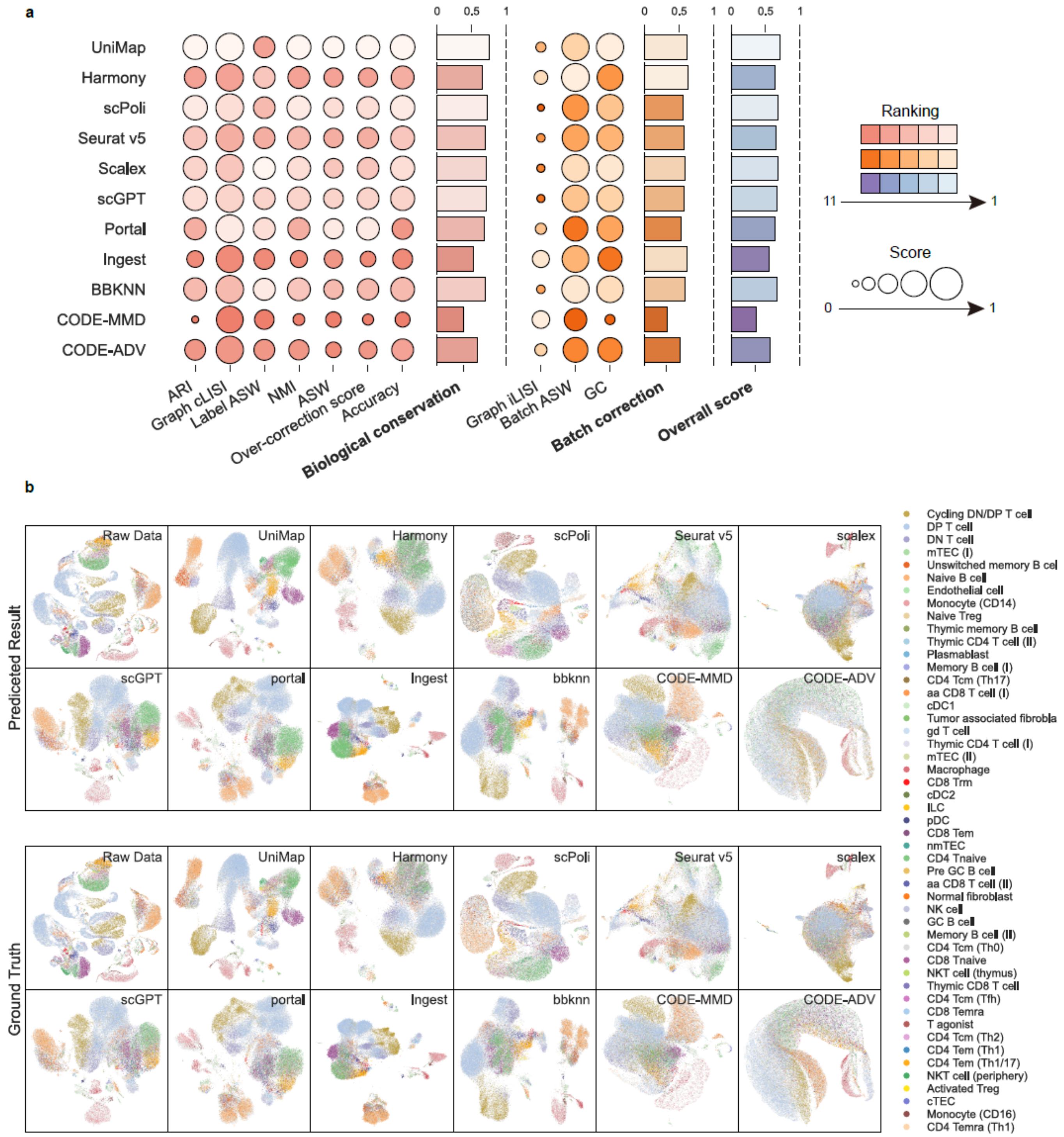

Figure S10. Comparisons of integration results of benchmark models on MG-part datasets. a) Overview of all benchmark models by overall score (purple) based on the MG-part datasets. Metrics are divided into biological conservation (red) and batch correction (orange). Overall scores are computed using the average of all individual metrics. All scores are normalized to a range of 0 to 1, with the higher values indicating better performance. b) UMAP plots showing the integration results of all benchmark models on the MG-part datasets, colored by predicted results and ground-truth labels.

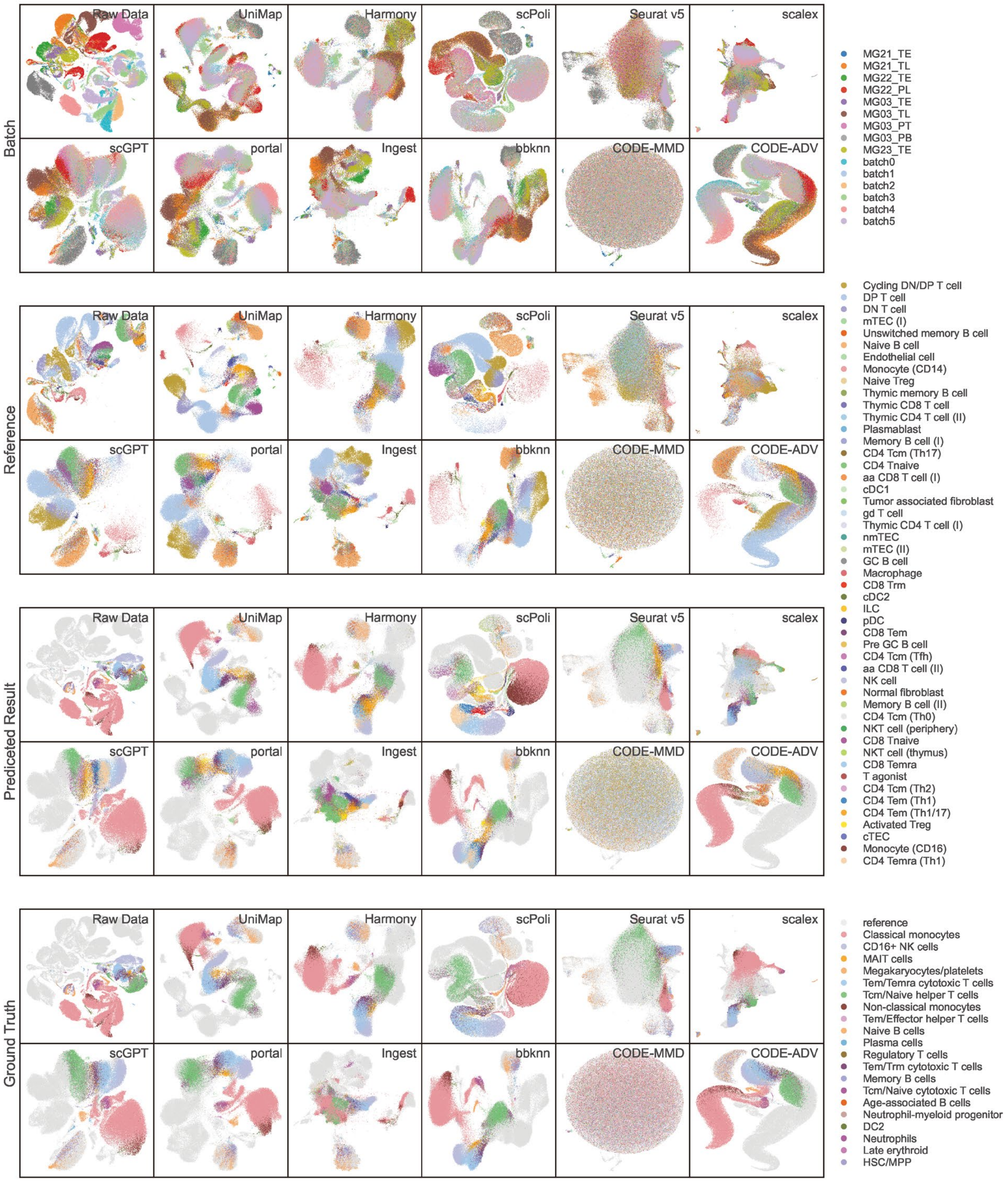

Figure S11. Comparisons of integration results of benchmark models on PBMC MG datasets. UMAP plots showing the integration results of all benchmark models on the PBMC MG datasets, colored by batches, predicted results, reference cell types, and ground-truth labels.

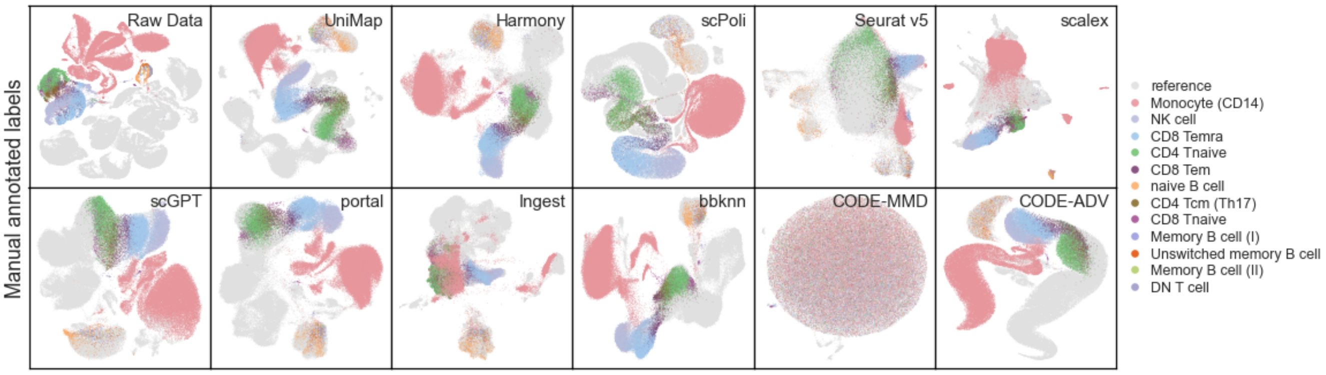

Figure S12. Comparisons of integration results of benchmark models on PBMC MG datasets (manual annotated labels). UMAP plots showing the integration results of all benchmark models on the PBMC MG datasets, colored by manual annotated labels.

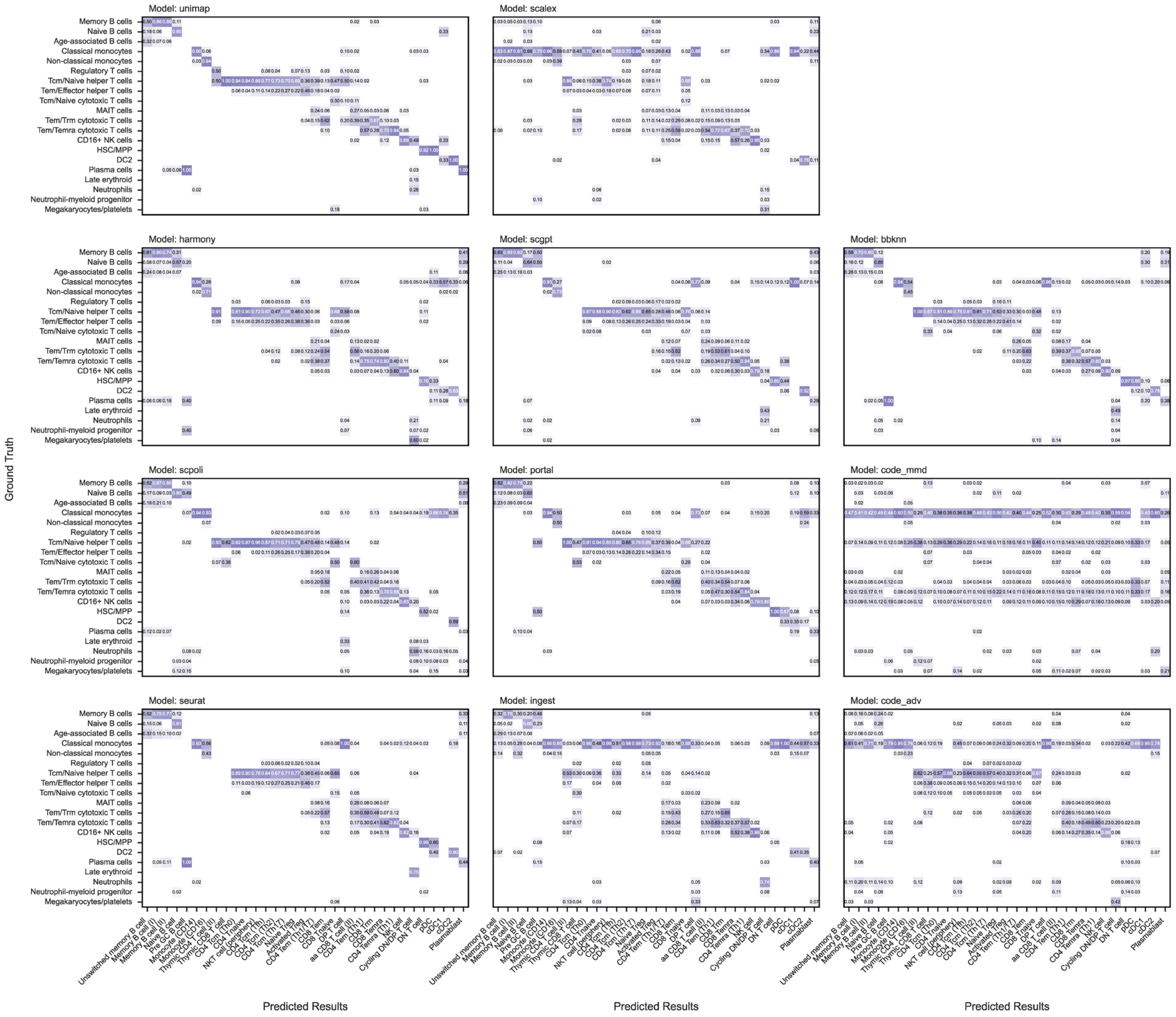

Figure S13. Comparisons of annotation results of benchmark models on PBMC MG datasets. Confusion matrixes showing the comparison of benchmark models in terms of predicted results on the PBMC MG datasets.

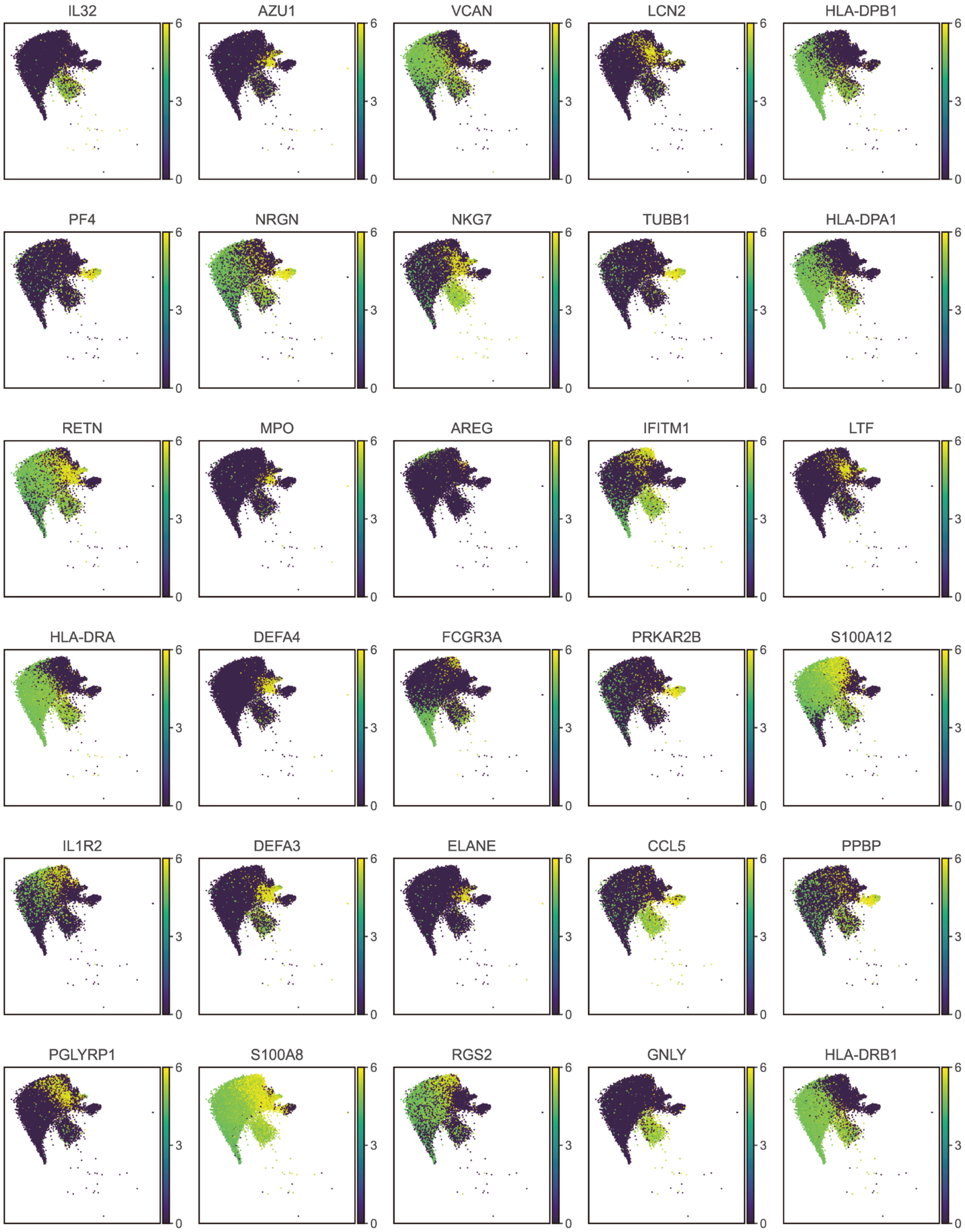

Figure S14. Canonical marker genes of monocyte population on the PBMC MG dataset 1. UMAP plots showing expression of differentially expressed genes by UniMap for cells predicted as monocytes on the PBMC MG dataset 1.

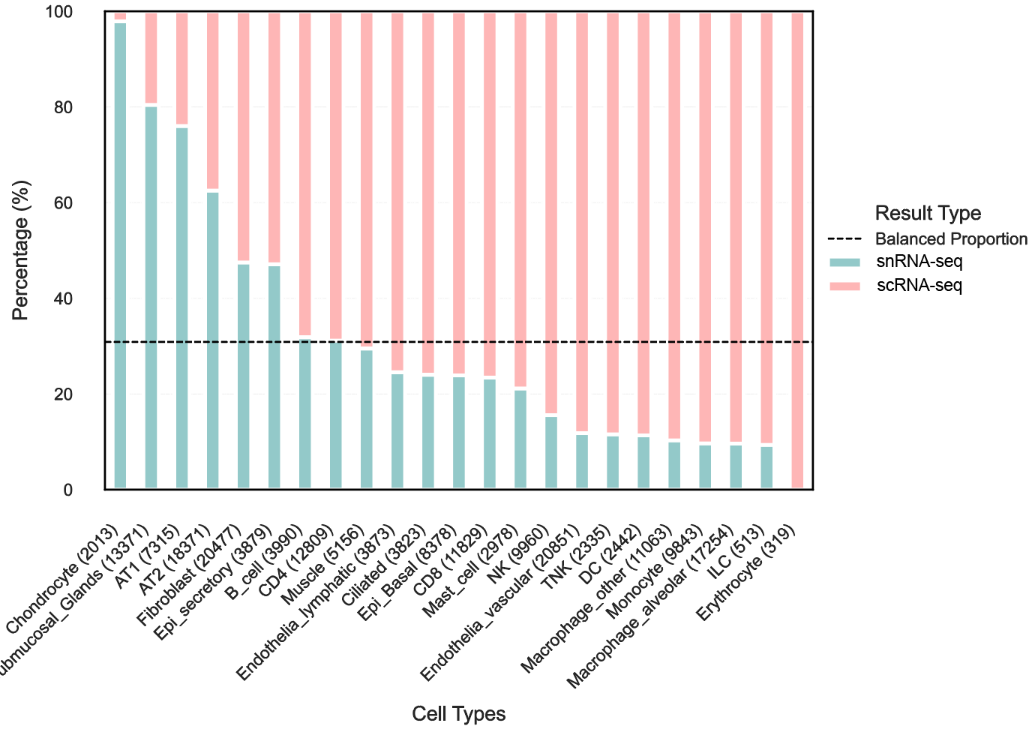

Figure S15. Distribution of Cell Types in the Lung dataset. Stacked bar plot showing the distribution of cell types across different sequencing methods from Lung dataset, with x-axis labels indicating the total cell count for each specific cell type in scRNA-seq and snRNA-seq.

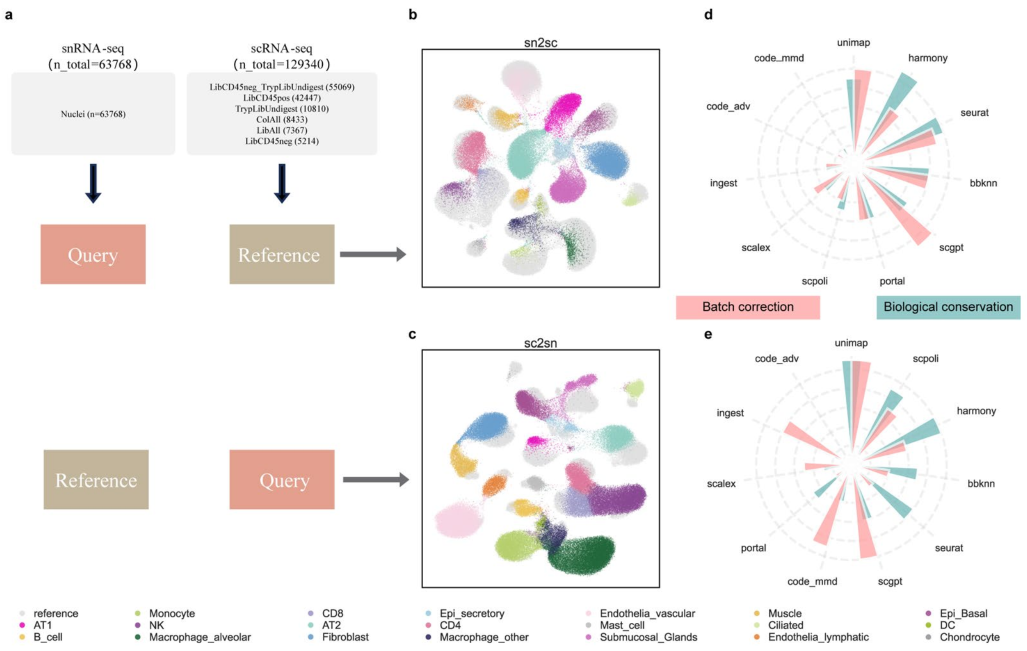

Figure S16. Predictive Performance and Benchmarking Analysis of UniMap Applied to the lung dataset. a) Schematic illustration of the annotation tasks (including batch information), showing bidirectional annotation between scRNA-seq and snRNA-seq data: using scRNA-seq as reference to annotate snRNA-seq, and using snRNA-seq to annotate scRNA-seq data. b,c) UMAP plots demonstrating UniMap's integration results on the Lung dataset, with cells colored by predicted labels (using snRNA-seq as query and scRNA-seq as query, respectively). d,e) Performance rankings of different benchmark models on the Lung dataset. Red bars represent batch correction scores while green bars indicate biological conservation scores, with longer bars corresponding to better rankings.

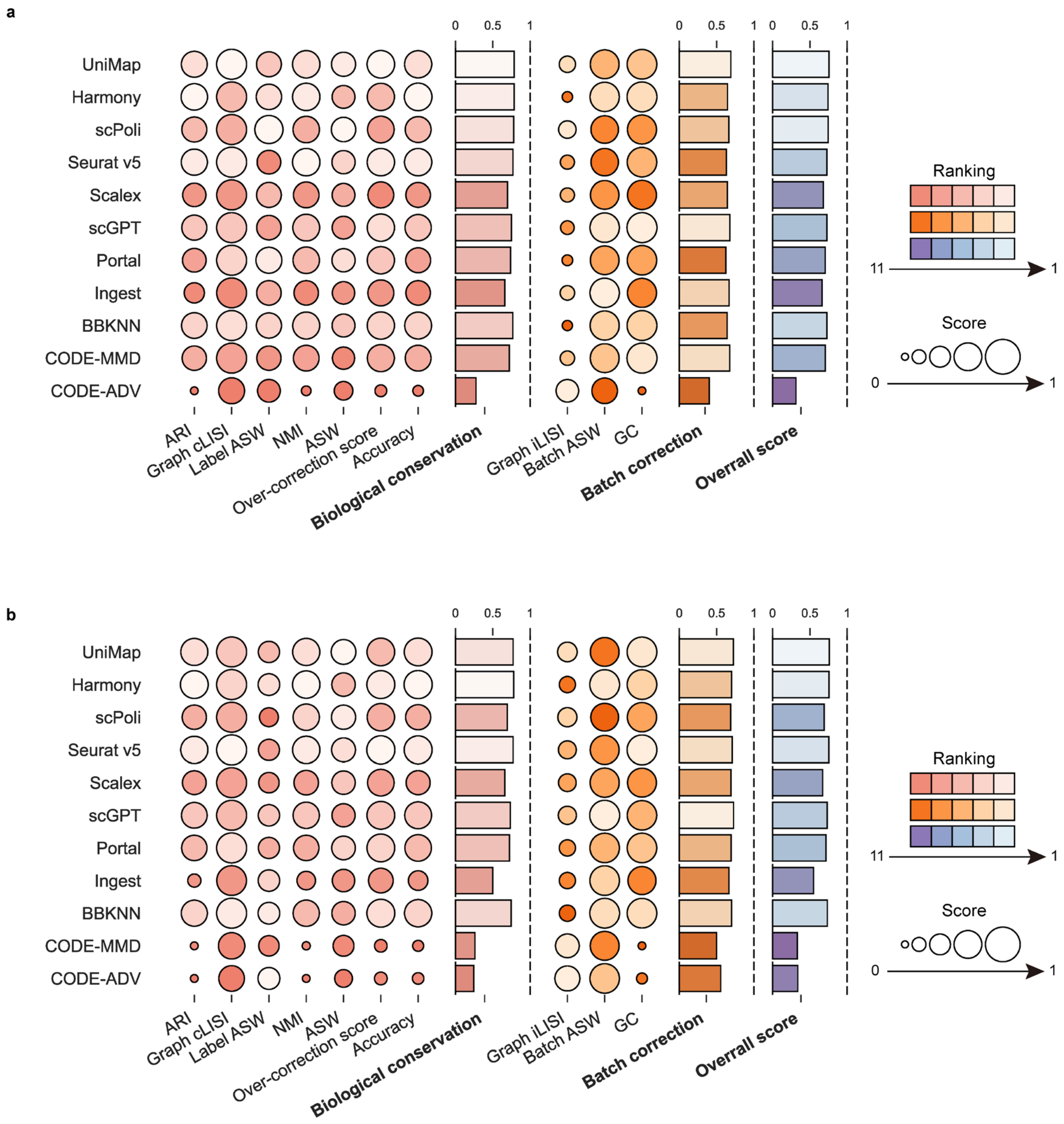

Figure S17. Comparisons of integration and annotation performance by quantification metrics. a,b) Overview of all benchmark models by overall score (purple) based on the LUNG dataset, where (a) uses snRNA-seq as reference to annotate scRNA-seq and (b) uses scRNA-seq to annotate snRNA-seq data. Metrics are divided into biological conservation (red) and batch correction (orange). Overall scores are computed using the average of all individual metrics. All scores are normalized to a range of 0 to 1, with higher values indicating better performance.

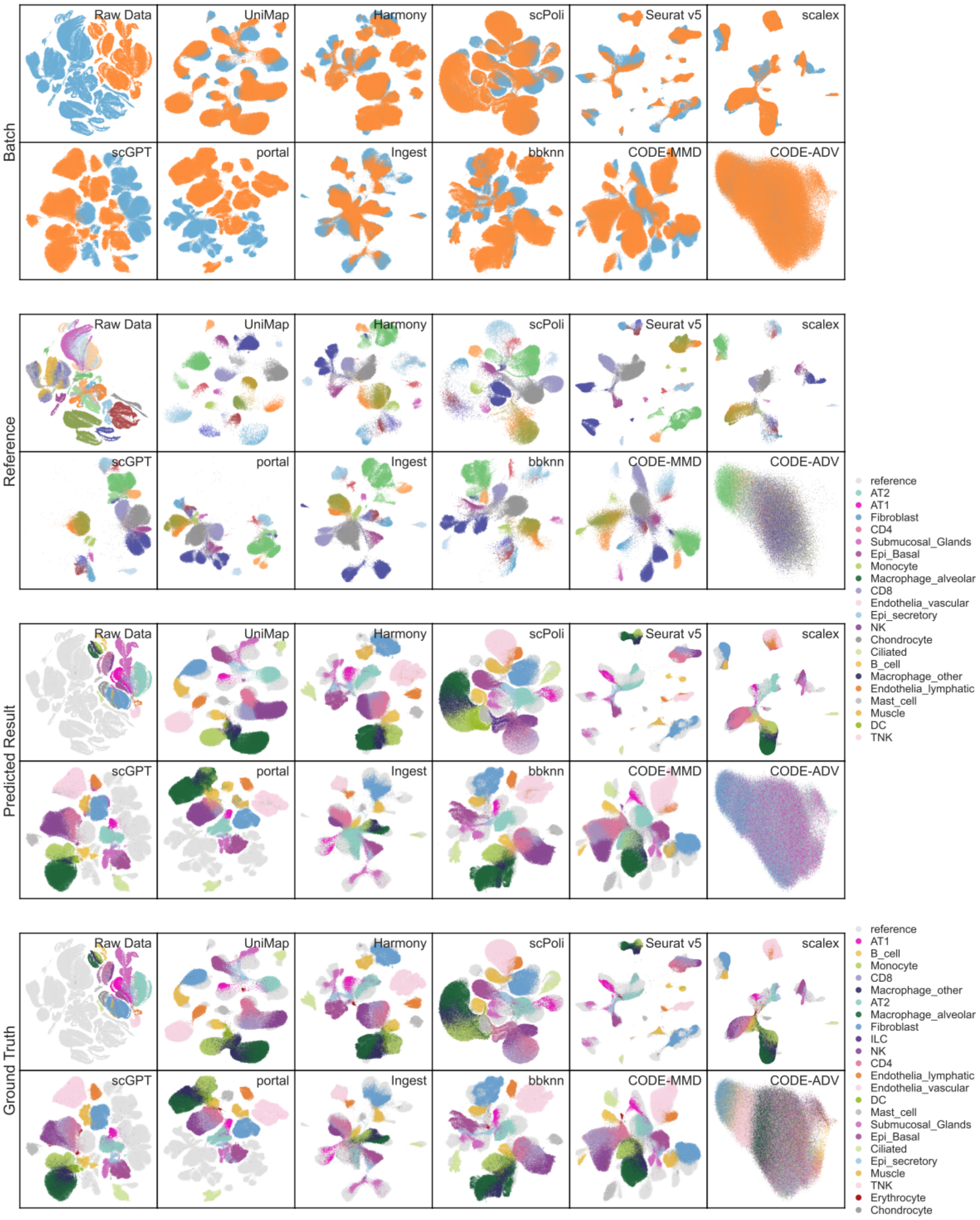

Figure S18. Comparisons of integration results of benchmark models on Lung datasets (using snRNA-seq as reference to annotate scRNA-seq). UMAP plots showing the integration results of all benchmark models on the Lung datasets using snRNA-seq as reference to annotate scRNA-seq, colored by batches, predicted results, reference cell types, and ground-truth labels.

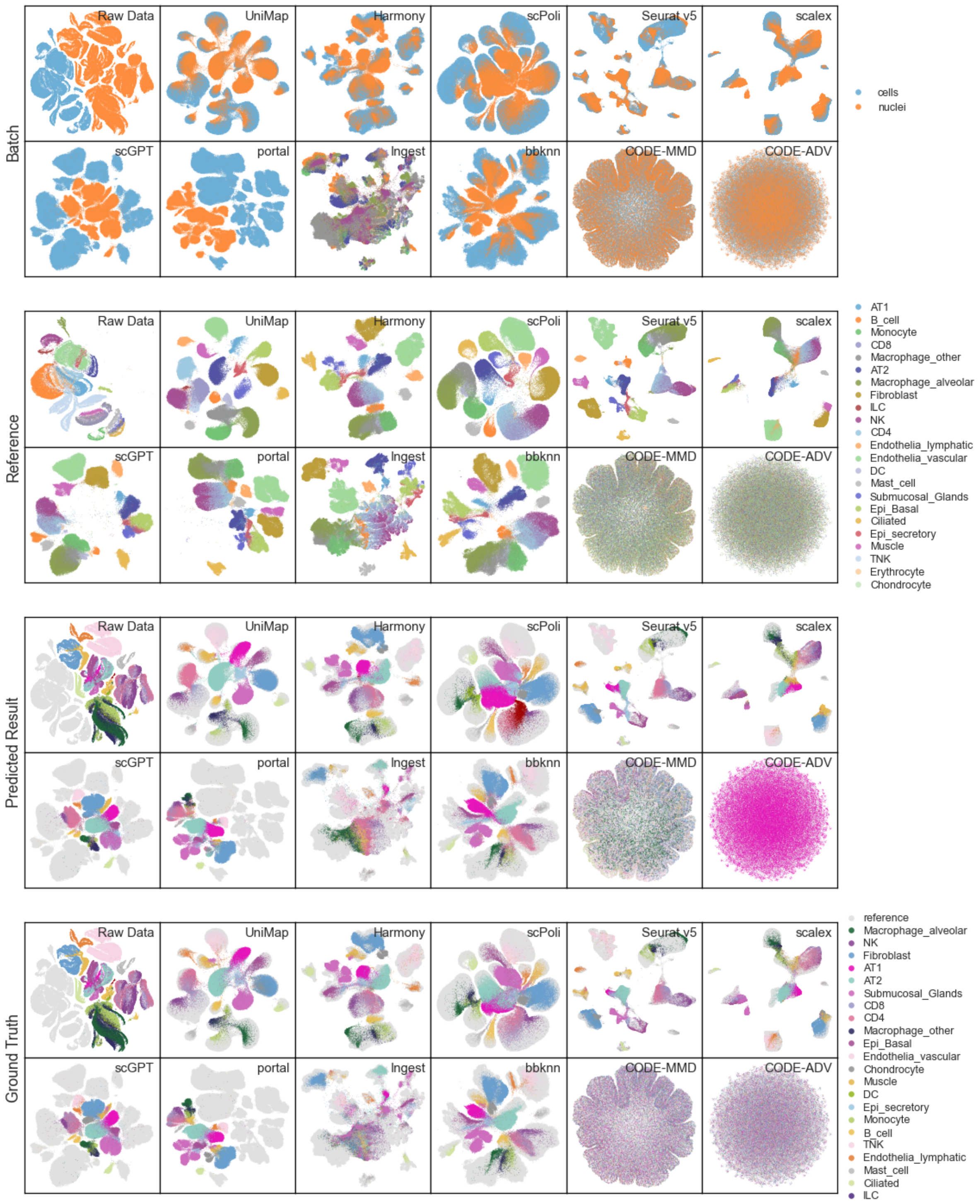

Figure S19. Comparisons of integration results of benchmark models on Lung datasets (using scRNA-seq as reference to annotate snRNA-seq). UMAP plots showing the integration results of all benchmark models on the Lung datasets using scRNA-seq as reference to annotate snRNA-seq, colored by batches, predicted results, reference cell types, and ground-truth labels.

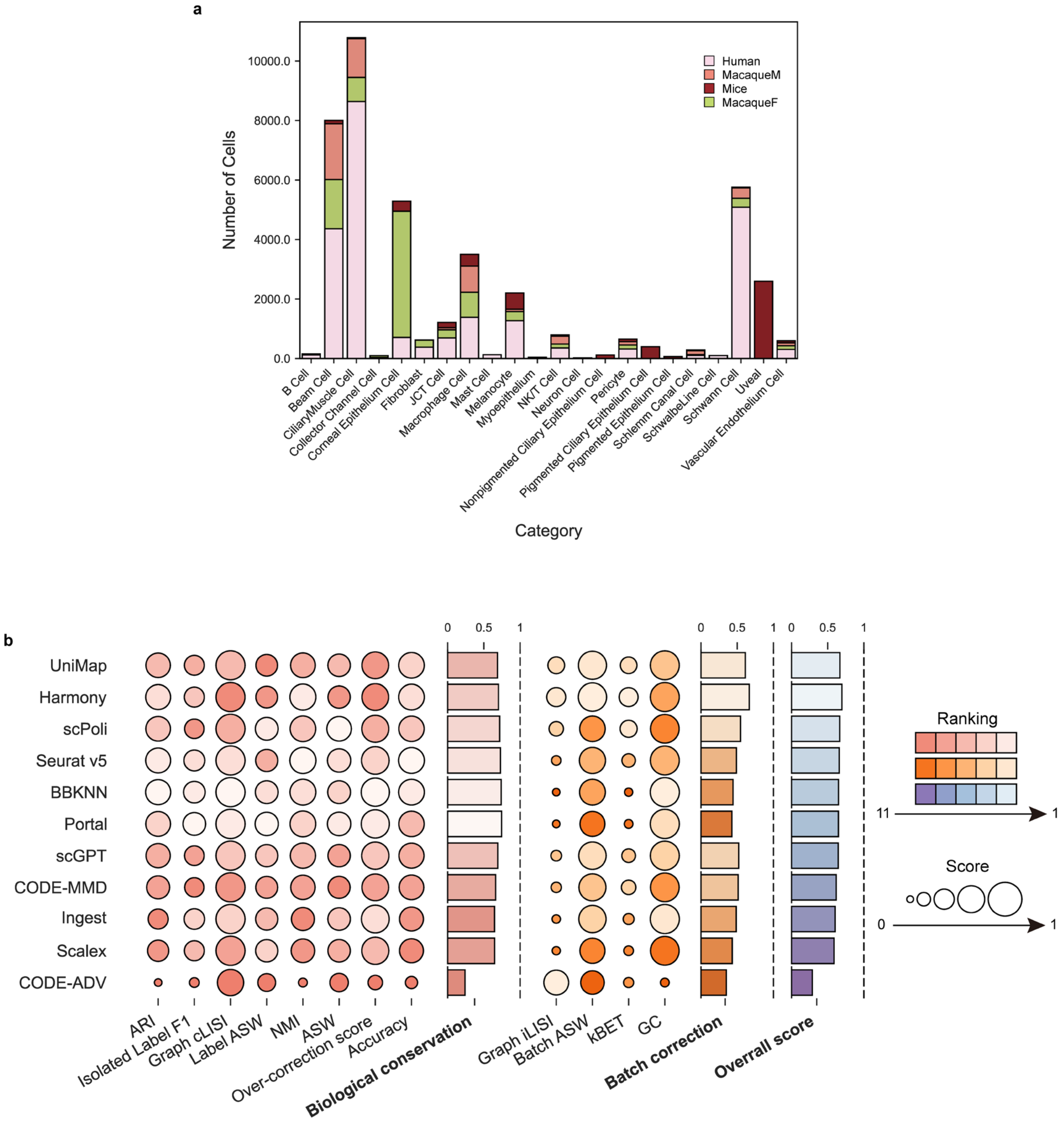

Figure S20. Composition of the Cross-species dataset and comparisons of integration performance by quantification metrics. a) Stacked bar plot showing the distribution of cell types across different species from Cross-species dataset. b) Overview of all benchmark models by overall score (purple) based on the Cross-species dataset. Metrics are divided into biological conservation (red) and batch correction (orange). Overall scores are computed using the average of all individual metrics. All scores are normalized to a range of 0 to 1, with the higher values indicating better performance.

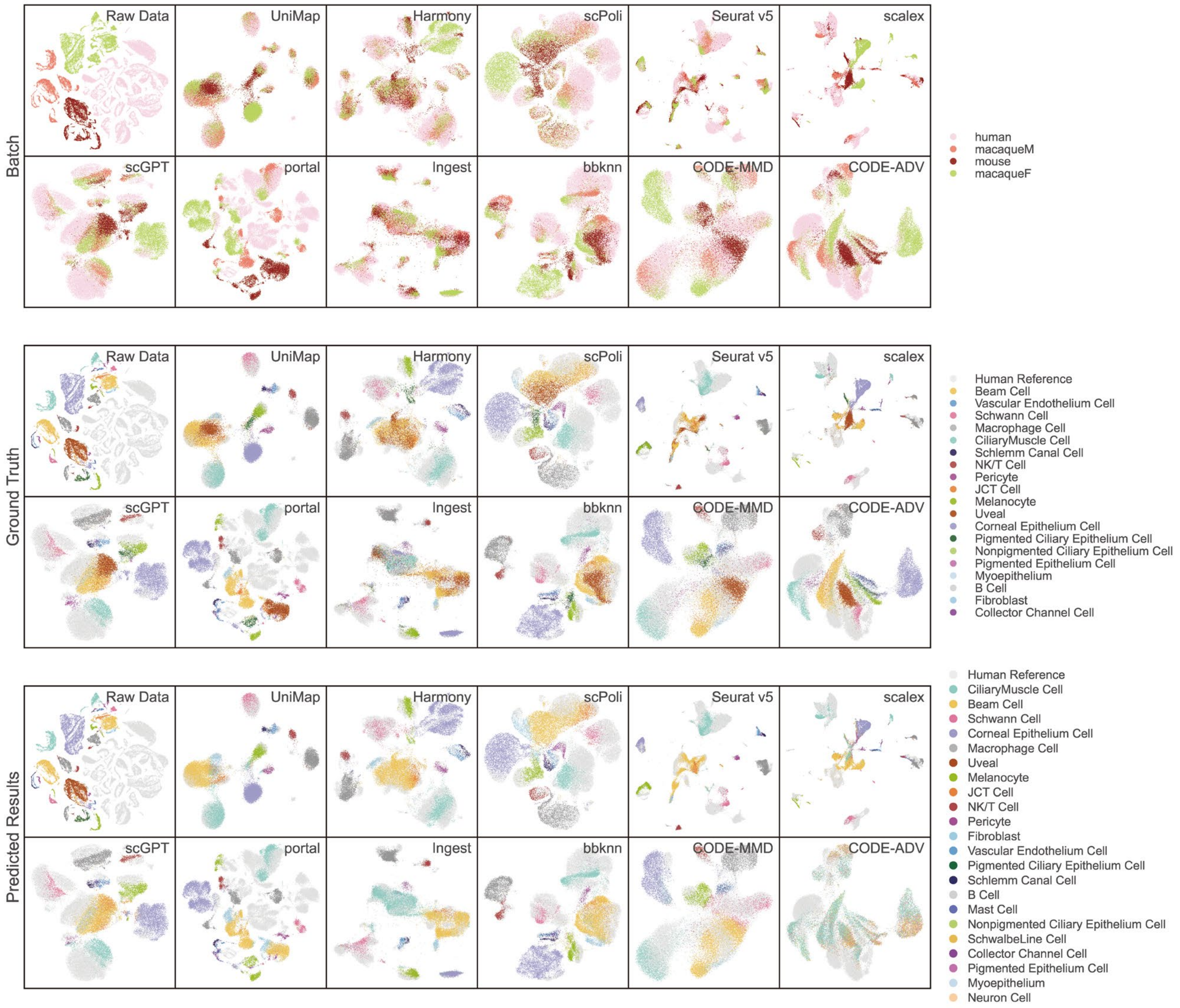

Figure S21. Comparisons of integration results of benchmark models on Cross-Species dataset. UMAP plots showing the integration results of all benchmark models based on the Cross-Species dataset, colored by batches, ground-truth labels and predicted results.

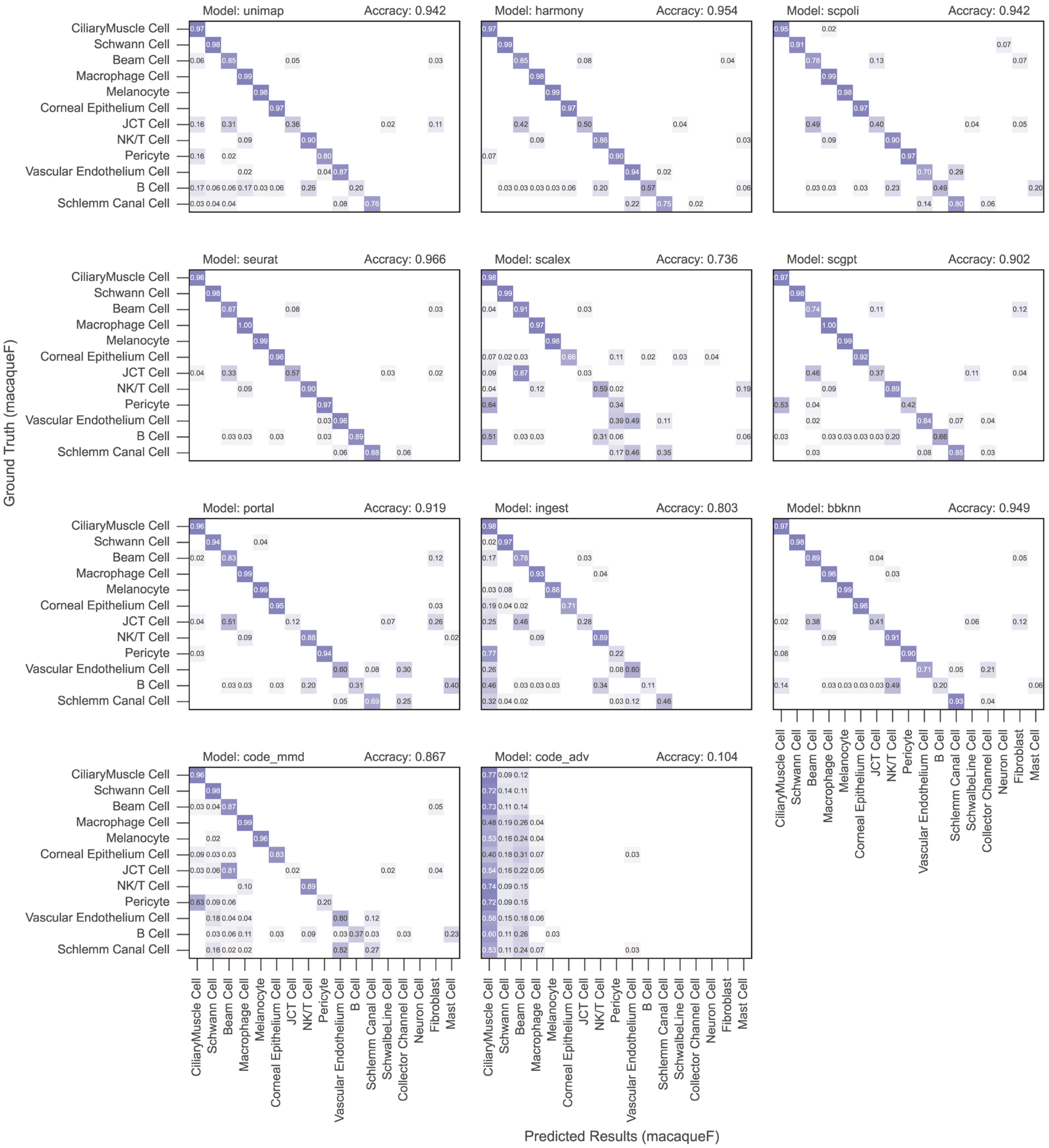

Figure S22. Comparisons of annotation results of benchmark models on Cross-Species dataset. a, Confusion matrixes showing the comparison of benchmark models in terms of predicted results from the MacaqueF species in Cross-Species dataset.

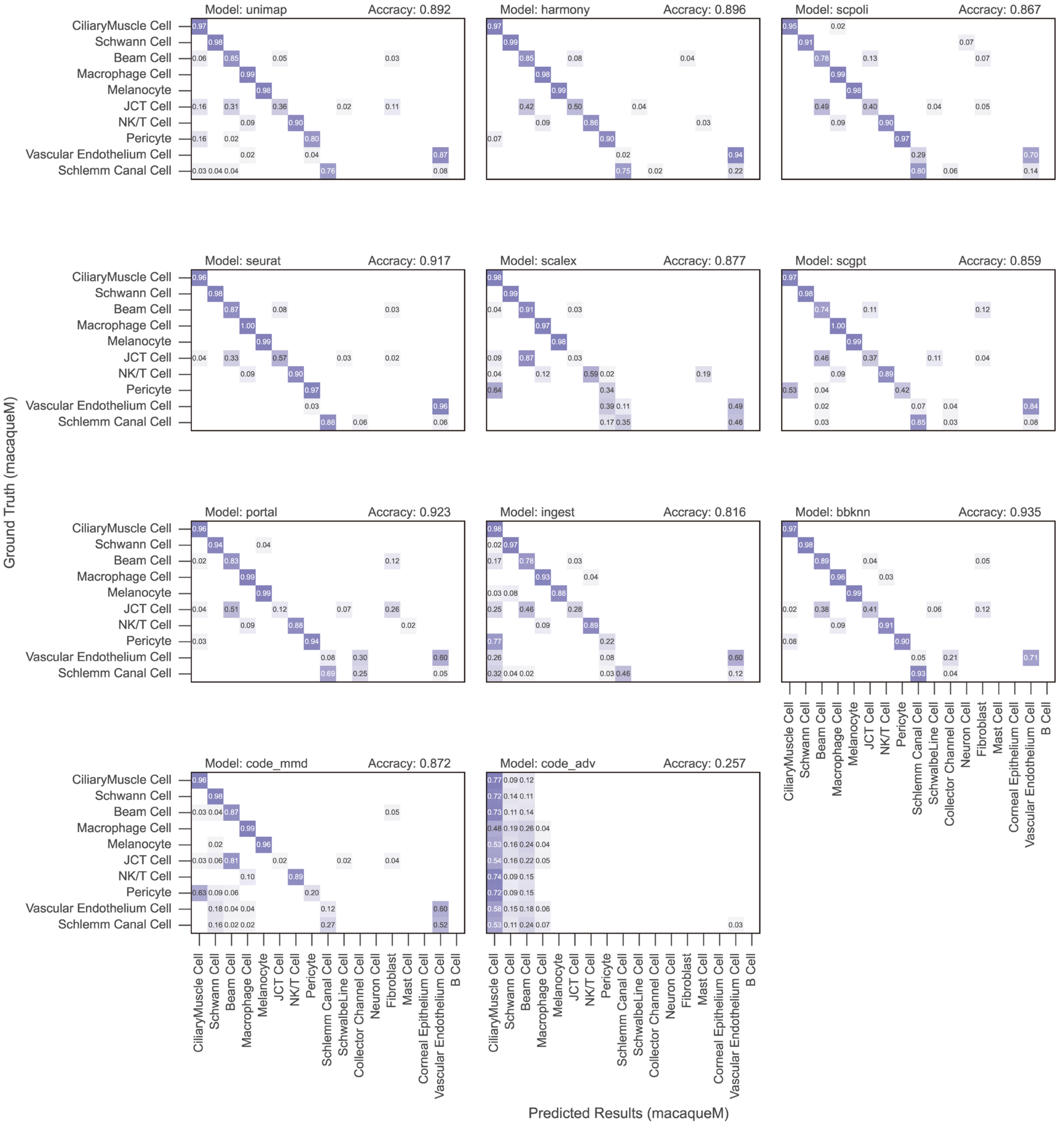

Figure S22. Comparisons of annotation results of benchmark models on Cross-Species dataset. b, Confusion matrixes showing the comparison of benchmark models in terms of predicted results from the MacaqueM species in Cross-Species dataset.

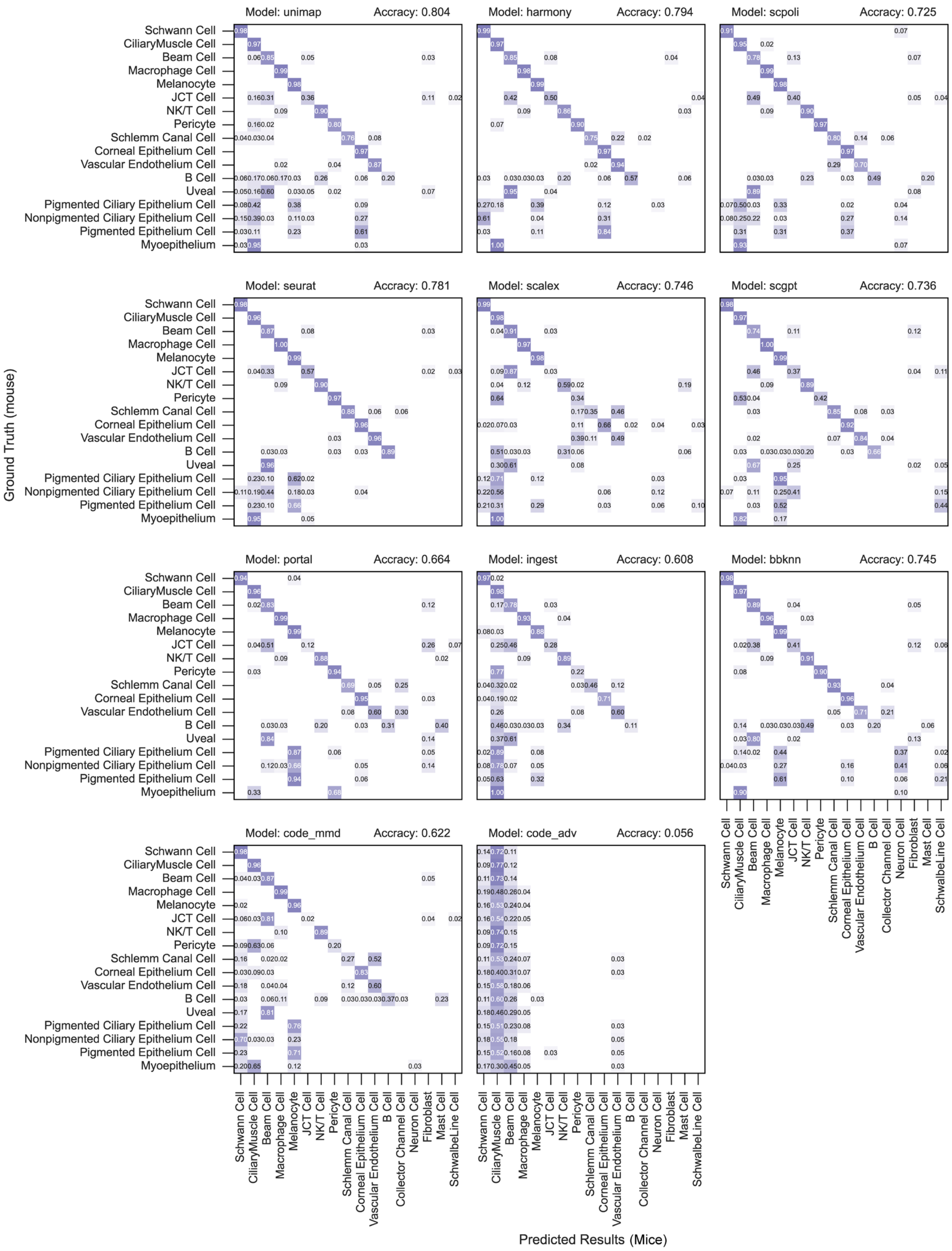

Figure S22. Comparisons of annotation results of benchmark models on Cross-Species dataset. c, Confusion matrixes showing the comparison of benchmark models in terms of predicted results from the mice species in Cross-Species dataset.

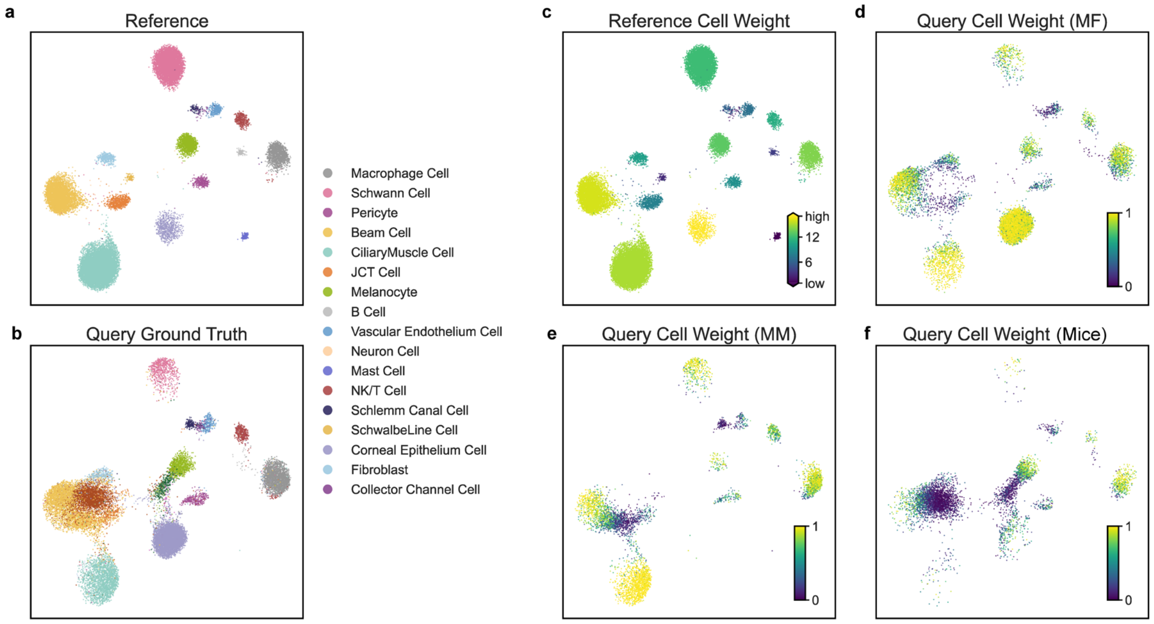

Figure S23. Comparisons of cell weights across different species. a, b) UMAP plot of integration result by UniMap on Cross-species dataset, colored by reference cell types and query ground-truth labels. c) UMAP plot of integration result by UniMap, colored by reference cell weight rankings. d-f) UMAP plot of integration result by UniMap, colored by cell weights for different species (MacaqueF, MacaqueM and Mice).

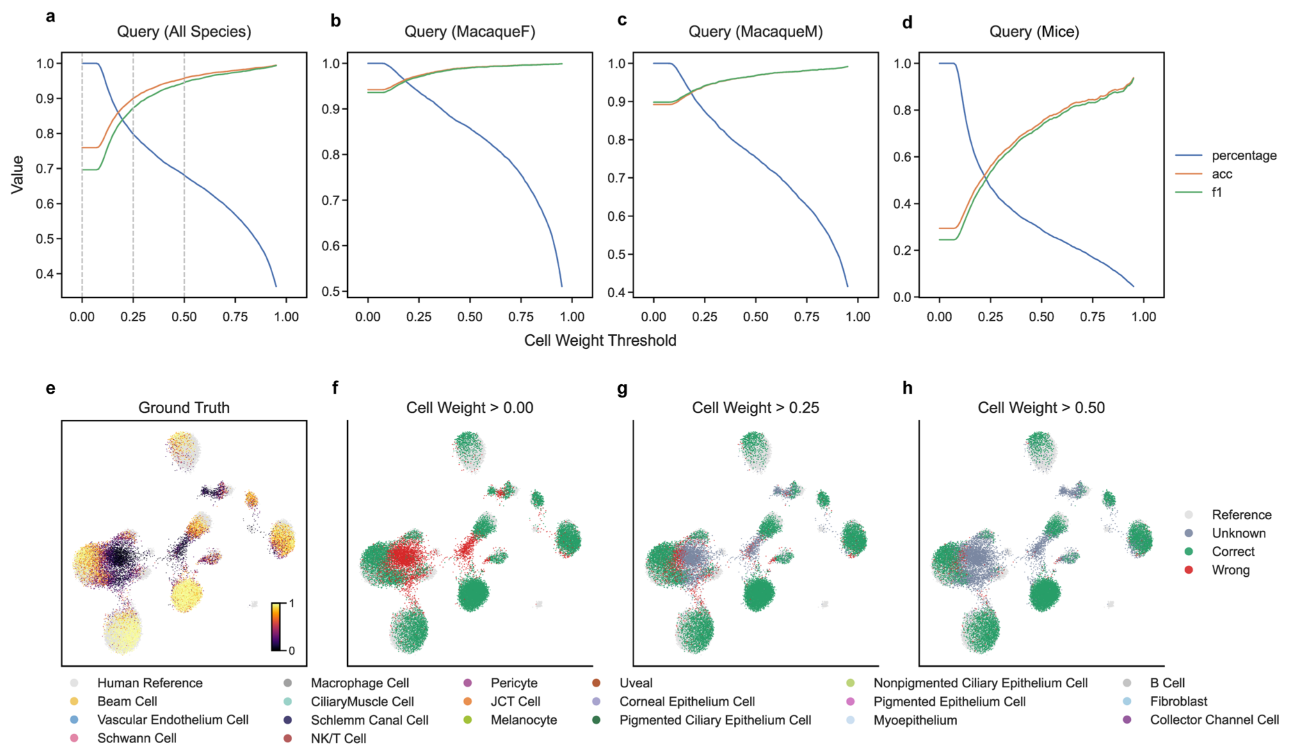

Figure S24. Impact of setting different cell weight thresholds on predicted results. a-d) Line plots showing the change trends of remaining cell proportion (blue), accuracy (orange), and F1 score (green) across different species (all species, MacaqueF, MacaqueM and mice) as the cell weight threshold increases. The y-axis represents the specific values of the metrics, and the x-axis represents the set of cell weight threshold. e) UMAP plot showing integration result of UniMap, colored by query cell weights. f-h) UMAP plots showing the results of setting different thresholds to classify cells with weights below that value as 'unknown' type.

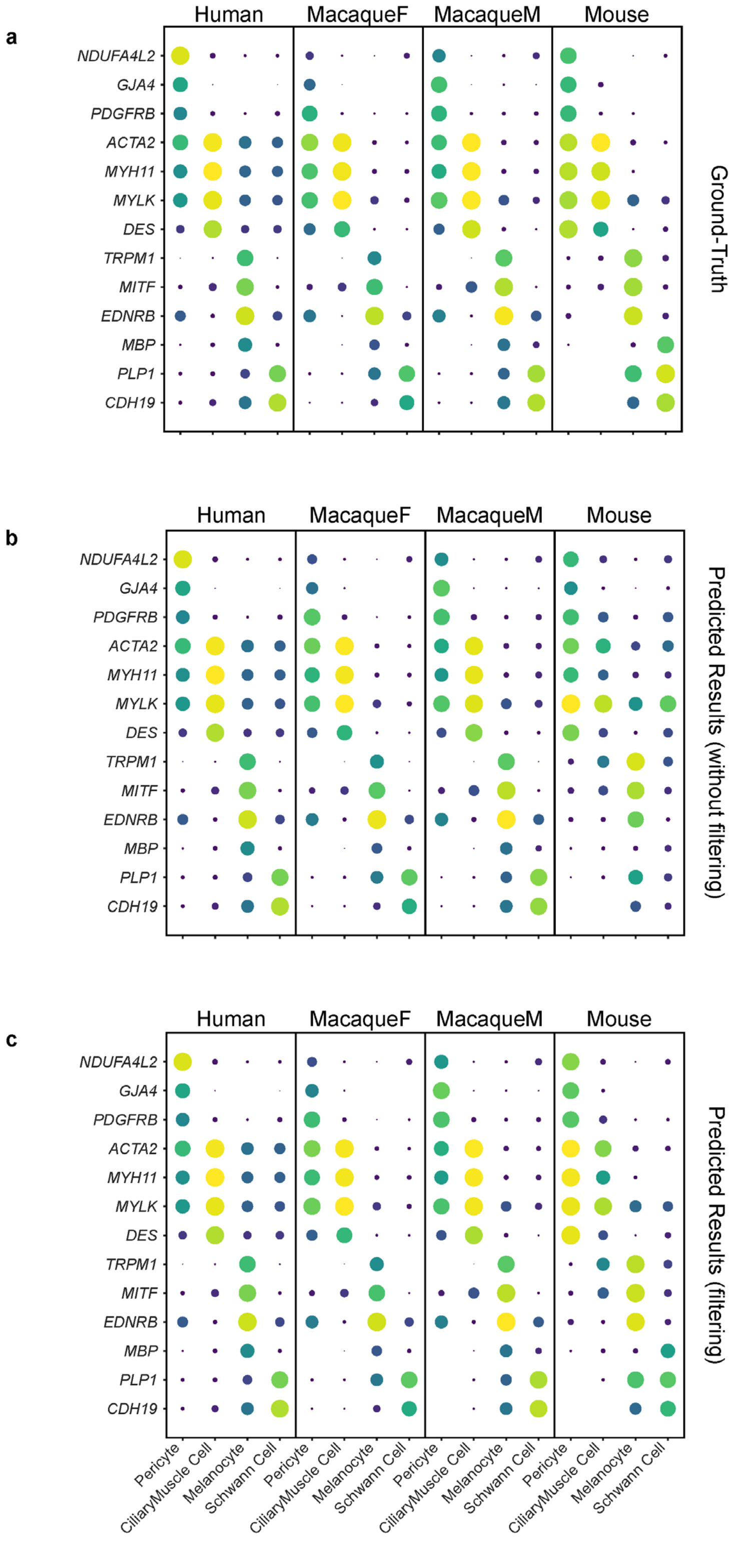

Figure S25. Comparison of gene expression patterns between ground-truth and predicted results. Dot plots showing expression of key genes in ground-truth (**a**), predicted results without fliting (**b**), and predicted results with fliting (**c**), with the color and size representing normalized gene expression and the percentage of cells expressing a given gene, respectively.

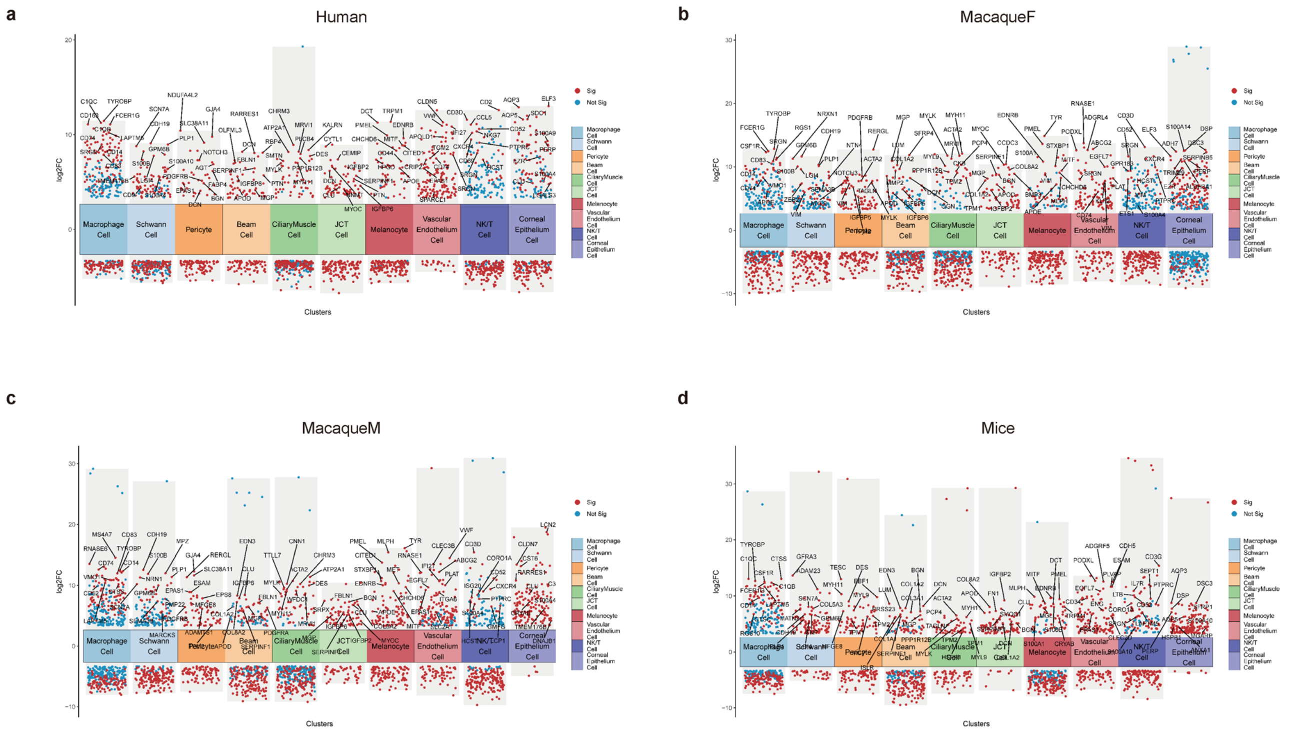

Figure S26. Marker genes identified across different species. The grouped volcano plots illustrating 100 representative highly variable genes for different predicted cell types across each species. Methodology involved grouping each species by its 10 major predicted celltypes and using the Wilcoxon rank-sum test to calculate log-fold change (logFC) and adjusted p-values for different groups. Genes with adjusted p-values less than 0.005 and the top 10 highest logFC rankings are selected as characteristic genes for this cell type in this species. Finally, the intersection of highly variable genes across all species forms the ultimate set of marker genes.

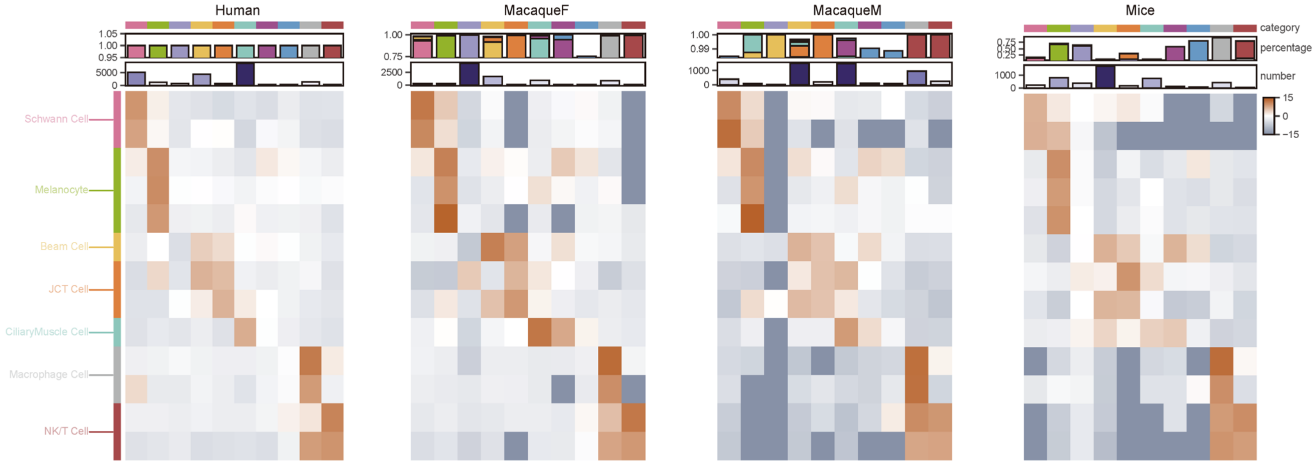

Figure S27. Comparison of gene expression patterns between different species. Average expression levels of 13 marker genes that are shared across four species for 10 major predicted cell types.

Table S1. Marker Genes for Manual Cell Type Annotation in the PBMC-COVID19 Dataset

Cell-type	Marker1	Marker2	Marker3	Marker4	Marker5
naive B cell	FCER2	TCL1A	IGHD	IGHM	IL4R
IgG plasma cell	IGHG1	IGHG2	IGHG3	-	-
dendritic cell, human	CD86	HLA-DRA	ITGAX	-	-
myeloid dendritic cell	CD1C	ITGAX	IRF4	CLEC9A	FLT3
plasmacytoid dendritic cell	CLEC4C	NRP1	TCL1A	IRF7	LILRA4
CD14-positive monocyte	CD14	S100A8	S100A9	VCAN	FCN1
CD14-low, CD16-positive monocyte	FCGR3A	CD52	IFITM2	LST1	TCF7L2
CD16-negative, CD56-bright natural killer cell, human	NCAM1	IL7R	KIT	XCL1	XCL2
CD16-positive, CD56-dim natural killer cell, human	FCGR3A	PRF1	GZMB	KLRF1	NCAM1
platelet	PPBP	PF4	GP1BA	GP9	ITGA2B
effector CD8-positive, alpha-beta T cell	CD8A	GZMB	PRF1	IFNG	NKG7
effector memory CD8-positive, alpha-beta T cell	CD8A	GZMK	ITGAE	SELL	CXCR3
central memory CD4-positive, alpha-beta T cell	CD4	CCR7	SELL	IL7R	LEF1
mature NK T cell	TRAC	TRBC1	CD3D	NKG7	GNLY
naive thymus-derived CD4-positive, alpha-beta T cell	CD4	CCR7	SELL	TCF7	LEF1
gamma-delta T cell	TRDC	TRGC1	NKG7	GNLY	IFNG
mucosal invariant T cell	TRAV1-2	NCAM1	IL18RAP	ZBTB16	SLC4A10
naive thymus-derived CD8-positive, alpha-beta T cell	CD8A	CCR7	SELL	IL7R	TCF7

Table S2. Marker Genes for Manual Cell Type Annotation in the MG Dataset

Cell-type	Marker1	Marker2	Marker3	Marker4	Marker5
Memory B cell (I)	JCHAIN	PLD4	ITM2C	ALOX5	CCR7
Memory B cell (II)	S100A10	ANXA2	ITGB1	CRIP2	S100A4
naive B cell	FCER2	TCL1A	IGHD	IGHM	IL4R
Unswitched memory B cell	CD1C	FCRL2	SYK	LY6E	FGR
Monocyte (CD14)	CD14	S100A8	S100A9	VCAN	FCN1
NK cell	GNLY	NKG7	TYROBP	KLRF1	GNLY
CD4 Tnaive	CCR7	NOG	SELL	AK5	TCF7
CD8 Tem	EOMES	GZMK	CXCR4	CXCR3	CD84
CD8 Temra	FCGR3A	NKG7	GNLY	KLRD1	CCL4
CD4 Tcm (Th17)	RORC	TNFRSF4	CCR6	LGALS1	ANXA2
CD8 Tnaive	CD8B	KLRK1	S100B	LRRN3	PASK
DN T cell	CDK6	IGLL1	DNTT	STMN1	NPM1

Table S3. Unified Naming Rules for Cross-Species Datasets

**Human**	**MF**	**Mice**	**MM**	**Final Label**
BCell	20_B cell	20_B cell		B Cell
BeamCella,<br>BeamCellb	2_BeamA<br>15_Beam X	14_Beam A<br>9_Beam Y	1_Beam X<br>4_Beam A	Beam Cell
CiliaryMuscle	4_Ciliary muscle	17_Ciliary muscle	2_Ciliary muscle	CiliaryMuscle Cell
CollectorChnlAqVein	14_Collector channel			Collector Channel Cell
				Corneal Endothelium
CornealEpi	1_Corneal epithelium<br>9_Corneal epithelium<br>17_Corneal epithelium<br>18_Corneal epithelium	5_Corneal epithelium<br>12_Corneal endothelium<br>13_Corneal		Corneal Epithelium Cell
Fibroblast	7_Fibroblast			Fibroblast
CribiformJCT	6_JCT	6_JCT	10_JCT	JCT Cell
Macrophage	3_Macrophage	4_Macrophage	3_Macrophage<br>15_Macrophage	Macrophage Cell
MastCell				Mast Cell
Melanocyte	8_Melanocyte<br>12_Melanocyte	2_Melanocyte	9_Melanocyte	Melanocyte
		15_Myoepithelium		Myoepithelium
NKT	10_NK/T cell	16_NK/T cell	6_NK/T<br>13_NK/T cell	NK/T Cell
Neuron				Neuron Cell
		7_Nonpigmented ciliary epithelium		Nonpigmented Ciliary Epithelium Cell
Pericyte	13_Pericyte<br>16_Pericyte	8_Pericyte	11_Pericyte<br>12_Pericyte	Pericyte
		3_Pigmented ciliary epithelium		Pigmented Ciliary Epithelium Cell
		11_Pigmented epithelium		Pigmented Epithelium Cell
ScEndo	19_SChlemm's Canal	19_SChlemm's Canal	7_SChlemm's Canal	Schlemm Canal Cell
SchwalbeLine				SchwalbeLine Cell
SchwannCell-my<br>SchwannCell-nmy	5_Schwann cell	18_Schwann cell	5_Nonmyelinating schwann cell<br>14_Myelinating schwann cell	Schwann Cell
		1_Uveal		Uveal
VascularEndo	11_Vascular	10_Vascular Endothelium	8_Endothelium	Vascular Endothelium Cell
